# Dissecting the influence of cellular senescence on cell mechanics and extracellular matrix formation in vitro

**DOI:** 10.1111/acel.13744

**Published:** 2022-12-13

**Authors:** Erik Brauer, Tobias Lange, Daniela Keller, Sophie Görlitz, Simone Cho, Jacqueline Keye, Manfred Gossen, Ansgar Petersen, Uwe Kornak

**Affiliations:** ^1^ Julius Wolff Institute Berlin Institute of Health at Charité ‐ Universitätsmedizin Berlin Berlin Germany; ^2^ Institute for Medical Genetics and Human Genetics Charité – Universtitätsmedizin Berlin Berlin Germany; ^3^ Flow & Mass Cytometry Core Facility Berlin Institute of Health at Charité – Universitätsmedizin Berlin Berlin Germany; ^4^ BIH Center for Regenerative Therapies (BCRT) Berlin Institute of Health at Charité – Universitätsmedizin Berlin Berlin Germany; ^5^ Institute of Active Polymers Helmholtz‐Zentrum Hereon Teltow Germany; ^6^ Institute of Human Genetics University Medical Center Göttingen Göttingen Germany

**Keywords:** cell force, cellular senescence, collagen, extracellular matrix, tissue regeneration, wound contraction

## Abstract

Tissue formation and healing both require cell proliferation and migration, but also extracellular matrix production and tensioning. In addition to restricting proliferation of damaged cells, increasing evidence suggests that cellular senescence also has distinct modulatory effects during wound healing and fibrosis. Yet, a direct role of senescent cells during tissue formation beyond paracrine signaling remains unknown. We here report how individual modules of the senescence program differentially influence cell mechanics and ECM expression with relevance for tissue formation. We compared DNA damage‐mediated and DNA damage‐independent senescence which was achieved through over‐expression of either p16^Ink4a^ or p21^Cip1^ cyclin‐dependent kinase inhibitors in primary human skin fibroblasts. Cellular senescence modulated focal adhesion size and composition. All senescent cells exhibited increased single cell forces which led to an increase in tissue stiffness and contraction in an in vitro 3D tissue formation model selectively for p16 and p21‐overexpressing cells. The mechanical component was complemented by an altered expression profile of ECM‐related genes including collagens, lysyl oxidases, and MMPs. We found that particularly the lack of collagen and lysyl oxidase expression in the case of DNA damage‐mediated senescence foiled their intrinsic mechanical potential. These observations highlight the active mechanical role of cellular senescence during tissue formation as well as the need to synthesize a functional ECM network capable of transferring and storing cellular forces.

## Graphical abstract

Cellular senescence modulates single cell mechanics and ECM formation with consequences for macroscopic tissue tensioning. Components of the SASP and a reduced collagen expression as a result of DNA damage‐mediated senescence competes with the increased mechanical potential of these cells.

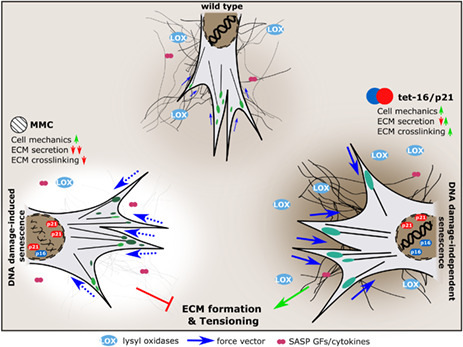

## 1 INTRODUCTION

Transient cell cycle inhibition in G1 or G2 phase is a key measure to prevent accumulation of genomic alterations. In contrast, replicative stress, DNA damage, and oncogene activation can induce long‐term cell cycle arrest that leads to cellular senescence. It is believed that in long‐lived multicellular organisms cellular senescence became a safeguard to prevent uncontrolled expansion of damaged and potentially malignant cells. Senescent cells secrete a variety of pro‐inflammatory cytokines as part of the senescence‐associated secretory phenotype (SASP) (Coppé et al., 2010) that lead to the recruitment of the immune system and subsequent cell clearance (Hoenicke & Zender, 2012; Kang et al., 2011). Senescent cells accumulate in aging organisms where their presence was shown to be detrimental as they compromise the functionality of surrounding cells and drives tissue degeneration through an increasing pro‐inflammatory milieu (Matjusaitis et al., 2016). Cellular senescence thus constitutes a hallmark of aging and is linked to various age‐related diseases such as cardiovascular diseases (Erusalimsky & Kurz, 2005), osteoarthritis (Martin & Buckwalter, 2003), and cancer (Campisi, 2013). Elimination of these cells can delay the onset of these diseases (Baker et al., 2011) and even revert age‐related tissue degeneration (Farr et al., 2017).

Various biomarkers can identify senescent cells (Matjusaitis et al., 2016) including senescence‐associated β‐galactosidase activity (SA‐βgal) (Debacq‐Chainiaux et al., 2009; Dimri et al., 1995) and the inhibitors of cyclin‐dependent kinase (CKI), p16^Ink4a^ and p21^Cip1^ (Krishnamurthy et al., 2004; Muñoz‐Espín et al., 2013). However, neither marker is senescence‐exclusive and can be triggered by alternative stimuli which makes it difficult to derive physiological consequences (Datto et al., 1995; Sharpless & Sherr, 2015; Untergasser et al., 2003). It is therefore suggested to combine multiple senescence markers to overcome the limited sensitivity and strong heterogeneity (Gorgoulis et al., 2019; Wiley et al., 2017).

For metazoan organisms, wound healing after trauma is an essential survival mechanism that ideally results in the re‐establishment of the original tissue integrity. It comprises several phases including inflammation, tissue formation, contraction, and remodeling. Early phases are characterized by the coordinated invasion of cells such as fibroblasts and myo‐fibroblasts (Hinz, 2010; Tomasek et al., 2002), followed by extracellular matrix (ECM) deposition and wound contraction. We recently provided insights into this mechanism by showing that macroscopic tissue tension is generated by a gradual transfer and storage of cellular forces into tensioned collagen fibers in a slip‐and‐ratchet mode (Brauer et al., 2019). In contrast to detrimental effects that result from the persistent presence of senescent cells, short‐term or transient senescence was shown to promote tissue remodeling during embryogenesis and wound healing (Da Silva‐Álvarez et al., 2020). Mechanistically, senescent cells were shown to drive myo‐fibroblast activation and wound closure through PDGF‐AA secretion as part of the SASP (Demaria et al., 2014). Yet, in the context of aging, elimination of accumulating p21‐positive cells during wound healing improved wound closure (Chia et al., 2021; Jiang et al., 2020). Other reports indicate that senescent cells appear during embryonic development through a p21/TGF‐β pathway‐dependent mechanism and contribute to tissue remodeling (Muñoz‐Espín et al., 2013) and exhibit anti‐fibrotic features in the development of liver cirrhosis (Krizhanovsky et al., 2008). Together, these observations sketch contradictory or at least context‐specific functions for tissue regeneration despite the uniform identification of these cells by the same markers. Whether such effects are purely mediated through paracrine effects or additionally through direct changes in cell mechanics and ECM synthesis and tensioning, is not yet fully understood.

Here, we investigated the role of cellular senescence on tissue formation and contraction using a recently established in vitro wound healing model (Brauer et al., 2019). Both, partial senescence (achieved by p16‐ or p21 over‐expression) and DNA‐damage mediated senescence modulated cellular adhesion, migration, and contractility with consequences for macroscopic, collective tissue formation, and tensioning in vitro. Our data highlight the mechanically active status of these cells and potentially a resulting beneficial role for tissue formation and tensioning beyond the canonical paracrine effect.

## 2 RESULTS

### 2.1 Modeling different types of cellular senescence in vitro

**FIGURE 1:**
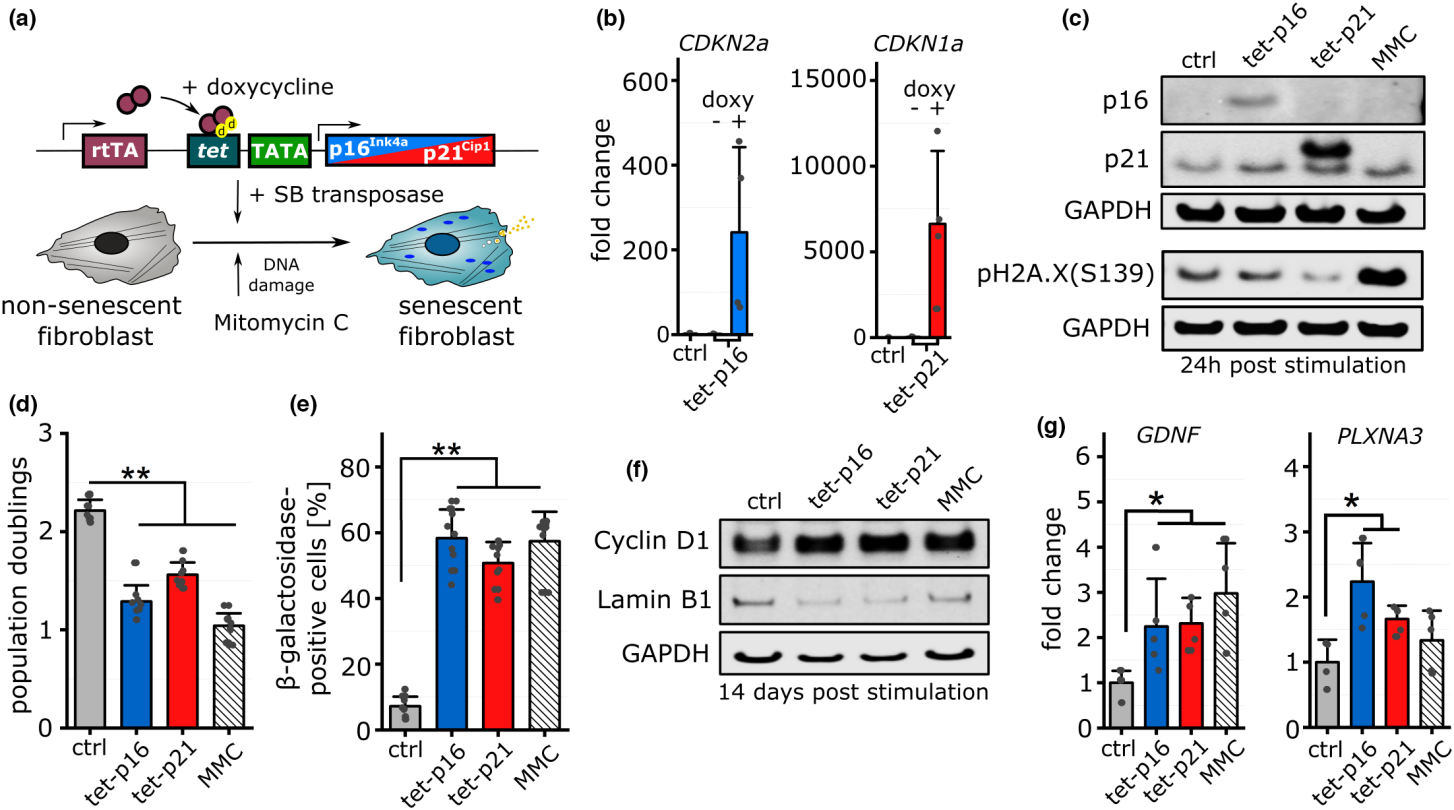
Validation of cellular senescence in vitro. (a) Schematic of the experimental setup to induce cellular senescence in primary human dermal fibroblasts. Tetracycline‐inducible expression constructs were stably integrated into the genome via co‐transfection with sleeping beauty (SB) transposase. Mitomycin C was used to drive cells into senescence through DNA damage response. (b) Gene expression (expressed as fold change relative to the control 24 post stimulation) of CDKN2a (p16^Ink4a^) and CDKN1a (p21^Cip1^) upon the addition of doxycycline (*n* = 4). (c) Immunoblotting and detection of p16, p21, and phospho‐H2A.X (Ser139) levels 24 h after induction (representative picture). GAPDH was used as loading control. (d) Proliferation of stimulated cells 7 days post induction of cellular senescence (*n* = 9) expressed as population doublings. (e) Percentile abundance of β‐galactosidase‐positive cells 14 days post induction of cellular senescence (*n* = 9). (f) Immunoblotting and detection of cyclin D1 and Lamin B1 levels 14 days after induction. GAPDH was used as loading control (representative picture). (g) Gene expression of glial cell line‐derived neurotrophic factor (GDNF) and plexin A3 (PLXNA3) 14 days after induction of senescence (*n* = 4–5).

We aimed at investigating the effect of canonical damage‐induced (DI) and partial, non‐damage‐induced (NDI) cellular senescence on tissue formation by primary human dermal fibroblasts known for their role in tissue repair processes. Over‐expression either of p16 or p21 (Althubiti et al., 2014; Capparelli et al., 2012; Helman et al., 2016) drives cells into cellular senescence without inducing a SASP (Coppé et al., 2011). Independent of cell damage as a triggering factor, these cells acquire characteristics of senescent cells. We either treated cells with the DNA crosslinking agent Mitomycin C (MMC) or established a cell cycle arrest through tetracycline‐inducible expression of p16 or p21 mediated by the tet‐transactivator, rtTA (Figure 1a, Figure S1a) (Gossen et al., 1995; Heinz et al., 2011).

In cells stably harboring the transposon‐based inducible expression constructs, specific, and conditional expression was validated after 24 h of doxycycline (DOX) stimulation that led to a strong upregulation of p16 and p21 transcript and protein levels (Figure 1b,c, Figure S1b). Analysis of H2A.X phosphorylation as a DNA damage marker revealed significant induction after MMC‐treatment but not after p16 or p21 over‐expression (Figure 1c, Figure S1b). In order to assess the percentages of cells reached by the treatments, we quantified the nuclear H2A.X phosphorylation (after MMC‐treatment) and p16/p21 protein expression (in transgenic cell lines treated by DOX). For all analyzed distribution profiles, we observed a range of 39%–56% positive cells compared with control (Figure S1c–e). We additionally measured the percentage of cells expressing the transgene by flow cytometry using a tet‐GFP control cell line that also showed 44% positive cells (Figure S1f). Our in vitro model is thus comparable to the natural in vivo situation, in which senescence as defined by the appearance of distinct markers, even at high ages, is only observed in a subset of cells in a given tissue (Michaloglou et al., 2005; Safwan‐Zaiter et al., 2022).

While cell proliferation was significantly reduced for all treated groups compared to control fibroblasts (Figure 1d, Figure S1g), SA‐β‐gal activity was significantly increased for DI and NDI senescence compared with control 7 and 14 days post stimulation (Figure 1e, Figure S1h,i). The percentage of SA‐β‐gal‐positive cells matched with the number of marker‐positive cells (H2A.X/FLAG, Figure S1c–e) mentioned before. As demonstrated before, only few genes reliably function as markers of the senescence phenotype (Hernandez‐Segura et al., 2017). Our analysis of selected markers either on transcript or protein level (Figure 1f,g, Figure S1g) revealed a significant upregulation of Cyclin D1, glial cell line‐derived neurotrophic factor (GDNF) and plexin A3 (PLXNA3). Lamin B1 levels were consistently downregulated as described before (Wang et al., 2017).

Taken together these data demonstrate, in accordance with previous reports that all three stimuli (tet‐p16/tet‐p21 (NDI), Mitomycin C (DI)) lead to the appearance of characteristic senescence‐related signatures.

### 2.2 Modulation of directional migration and adhesion through cellular senescence

**FIGURE 2:**
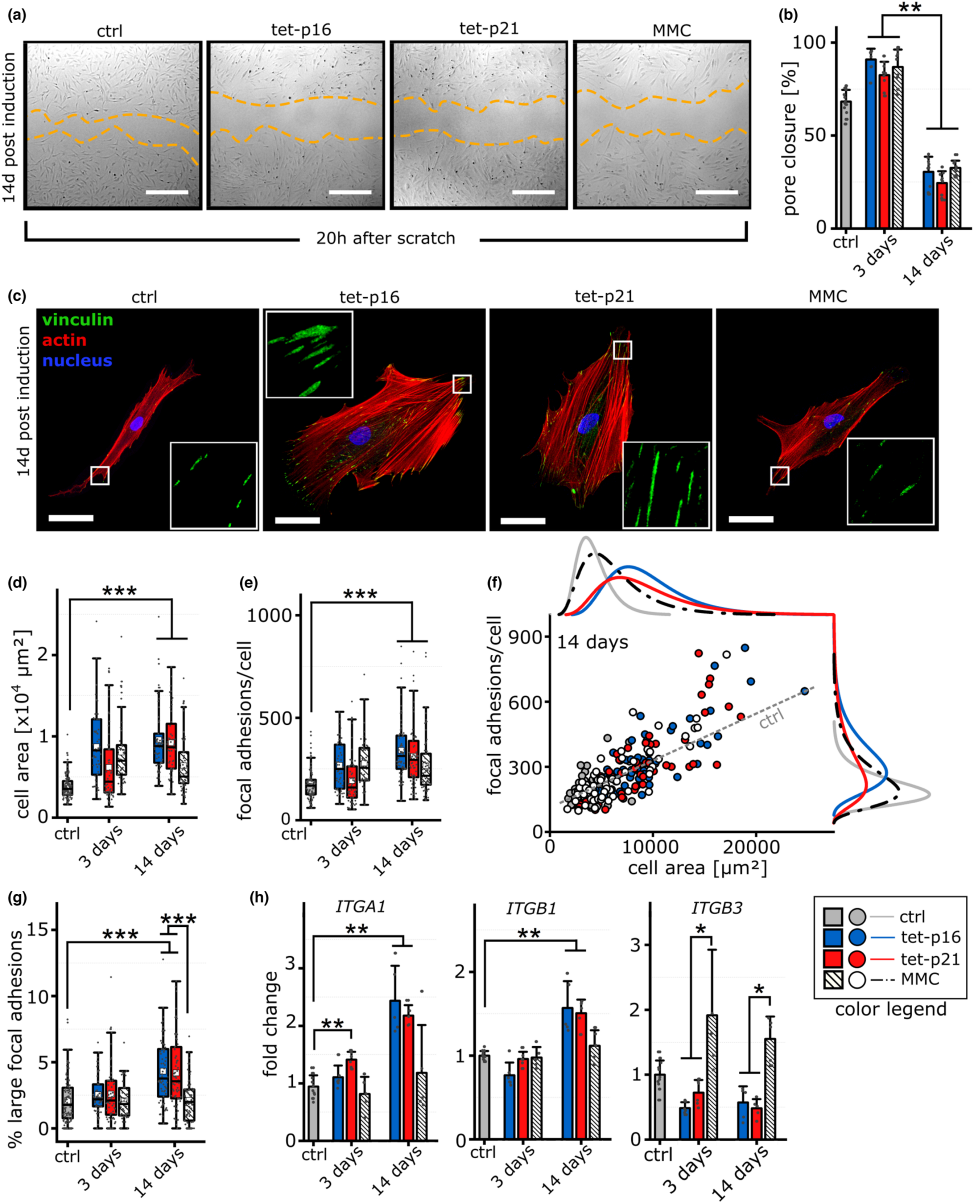
Modulation of cell migration, morphology & adhesion. (a) Representative images of scratch wound healing 20 h post scratch. Scale bar 200 μm. (b) Quantification of pore closure 20 h post scratch (*n* = 6–7). (c) Representative images 14 days post induction of cellular senescence. Cells were stained for focal adhesion marker vinculin (green), Actin cytoskeleton (red) and cell nuclei (blue). Scale bar 50 μm. (d) Quantification of single cell area 3 and 14 days postinduction of cellular senescence (*n* ≥ 50 cells). (e) Quantification of focal adhesions (absolute count) per cell (*n* ≥ 50 cells) 3 and 14 days after induction of senescence. (f) Correlation of focal adhesion count per cell (y axis) and cell area. Each colored dot represents a single cell and marginal log‐normal distribution curves to indicate the population distribution. Grey dashed line indicates extrapolated linear regression of control cells. (g) Percentile amount of large focal adhesions (>5 μm^2^) (*n* ≥ 50 cells). (h) Gene expression of integrin alpha 1 (ITGA1), integrin beta 1 (ITGB1) and integrin beta 3 (ITGB3) 3 and 14 days post induction (*n* = 4–5).

In order to understand how senescence interferes with the cellular capacity for directional migration, we performed scratch wound assays 3 and 14 days post induction of senescence (Figure 2a,b) (Pumberger et al., 2016). Three days after induction of senescence, the migratory potential was mildly enhanced compared with controls. However, 14 days after induction, gap closure was strongly reduced for all senescent groups. This suggests that shortly after induction senescent cells retain their motility which ceases during the course of senescence manifestation.

Differences in the migratory capacity might be caused by altered focal adhesion (FA) dynamics (Kim & Wirtz, 2013). We thus analyzed cell and FA morphology by confocal microscopy. Tet‐p16 or tet‐p21 cells showed a strongly enlarged cell size (higher cell area) and more rounded (decreased aspect ratio) morphology which was visible, but less pronounced for MMC‐treated cells (Figure 2c,d, Figure S2a). Vinculin staining revealed that in particular tet‐16 and tet‐p21 cells exhibited a significantly increased FA count both after 3 and 14 days (Figure 2e). At the same time, tet‐p16 and tet‐p21 and to a lesser extend also MMC treatment resulted in a shift of the population toward cells with both, larger cell area and higher number of FAs (Figure 2f). A closer look revealed a time‐dependent maturation towards large FAs for tet‐p16 and tet‐p21 cells with a slight increase in the number of medium‐sized FAs (1–5 μm^2^) after 3 days (Figure S2c) and a significantly higher number of large FAs 14 days after induction compared with control cells (Figure 2g). Small (0.25–1 μm^2^) or tiny (<0.25 μm^2^) FAs revealed only minor changes (Figure S2d,e). Such a FA maturation was not observed for MMC‐treated cells.

As the combination of integrin alpha and beta isoforms determines the ECM binding motif, we analyzed the expression levels of selected integrin receptors in order to unravel differences in FA composition due to senescence. Tet‐p16 and tet‐p21 cells showed increased transcript levels of alpha 1 and beta 1 integrins relative to control cells after 14 days, which was not visible after 3 days (Figure 2h). Expression levels of other integrins such as alpha 5, alpha V and beta 5 were largely unresponsive to senescence‐triggering interventions for all analyzed time points (Figure S2h). Intriguingly, beta 3 integrin expression was downregulated upon p16 or p21 over‐expression and upregulated for MMC‐treated cells for both analyzed time points (Figure 2h). Beta 3 integrin was recently described as a marker of cellular senescence (Rapisarda et al., 2017).

Together, these data demonstrate that senescence affects cellular morphology and substrate anchoring in a time‐dependent manner. In particular, p16 and p21 over‐expression resulted in an increased abundance of large FAs. Although DI‐senescence resulted in an enlarged size and higher focal adhesion counts, the FA size distribution pattern was only mildly altered. These potentially affect cellular mechano‐sensation, traction, and 3D ECM formation.

### 2.3 The senescence program affects cell mechanics

**FIGURE 3:**
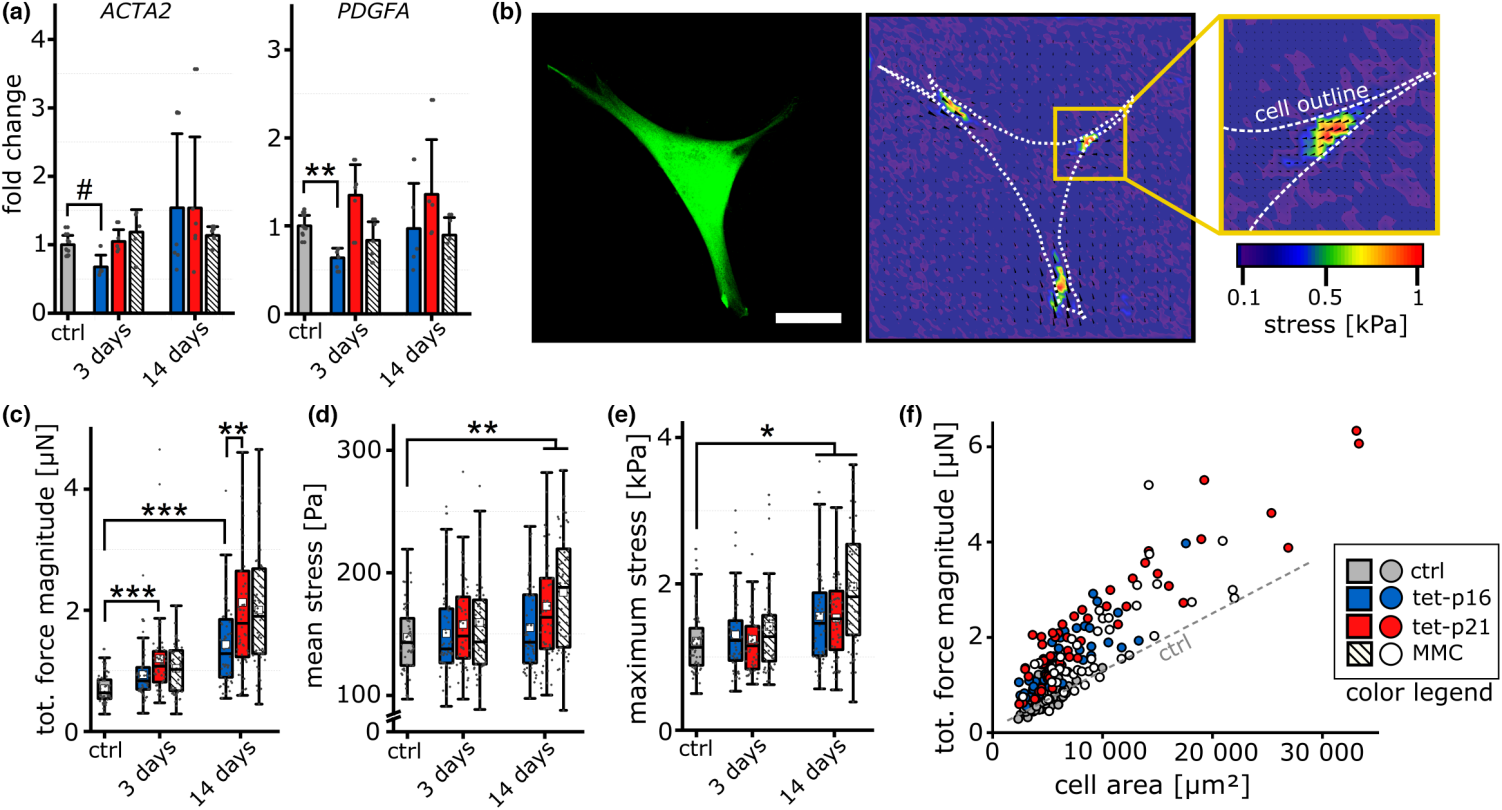
Cellular senescence increases single cell forces. (a) Gene expression levels for ACTA2 and PDGFA genes 3 and 14 days after stimulation. (b) Representative image of a fibroblast (control) seeded onto PAA gels. Scale bar 50 μm. Right: Force magnitude map overlaid with displacement vectors (white arrows). (c) Total force magnitude 3 and 14 days after induction of cellular senescence (*n* > 50). (d and e) Mean and maximum stress recorded for individual cells (*n* > 50). (f) Correlation of total force magnitude and cell area 14 days after stimulation. Dashed lines indicate linear regression for control cells.

We speculated that altered cell morphology and FA characteristics of senescent cells affected their single cell mechanics. It was reported that senescent cells secrete PDGF‐AA which drives myo‐fibroblast activation and consequently wound contraction (Demaria et al., 2014). We therefore analyzed the expression of alpha smooth muscle actin (aSMA) and PDGF‐AA after 3 and 14 days (Figure 3a). However, we did not observe a consistent or significant upregulation of either genes, but even a mild down‐regulation after 3 days post induction for tet‐p16.

We subsequently performed traction force microscopy (TFM) to monitor single cell forces 3 and 14 days after senescence induction (Figure 3c, Figure S3a). After 3 days, we already observed a slight increase in total force magnitude for all treatments compared with the control which was significant particularly for tet‐p21 cells (Figure 3b). This increase was further pronounced at Day 14 and intriguingly, tet‐p21 cells showed an even enhanced total force magnitude compared to tet‐p16 cells. Furthermore, after 14 days both the mean stress increased for tet‐p21 and MMC groups and the maximum stress for all senescence stimuli compared to control cells (Figure 3c,d). This was surprising since ACTA2 transcript levels would not have suggested an increased contractility of senescent cells, particularly MMC‐treated. However, it has to be noted that TFM measurements were performed on soft substrates (15 kPa) while ACTA2 transcript levels were measured on plastic. As differences in substrate stiffness were reported before to influence cellular mechanical activity (Elosegui‐Artola et al., 2016), we cultured control and MMC‐treated cells on soft PDMS substrates (1 kPa). Indeed, we observed slightly higher alpha‐SMA levels for MMC compared to control cells (Figure S3b,c). While this validates our TFM data, it further indicates a different mechano‐responsiveness of MMC‐treated cells as the alpha‐SMA levels were less regulated by stiffness compared with control cells. Since senescent cells exhibit an enlarged size, higher total forces are required to ensure stable average stresses. The presented data yet indicate an over‐proportional increase beyond a mere compensation for an enlarged cell size. This was reflected in the correlation of cell area and total force magnitude where most senescent cells revealed an over‐proportional increase in the total force magnitude compared to control (Figure 3f).

Taken together, TFM revealed a dynamic regulation and consistent increase of single cells forces for all senescence groups—tallying with the observations made for focal adhesion morphology (Figure 2). As observed for focal adhesion morphology, this mechanical activation was time‐dependent and slowly emerged over the investigate time period of 14 days.

### 2.4 Consequences of senescence‐related mechanical activation for tissue formation and contraction

The quantification of single cell forces indicated a time‐dependent variation and progressive increase in cellular contractility after senescence induction. In this context, we recently demonstrated that the generation of macroscopic tension in 3D tissue greatly depends on the ability to synthesize a load‐bearing fibrillar collagen network (Brauer et al., 2019). Hence, macroscopic tissue formation is regarded to be controlled by an interlay of ECM deposition and ECM tensioning through cell traction forces applied via FAs.

**FIGURE 4:**
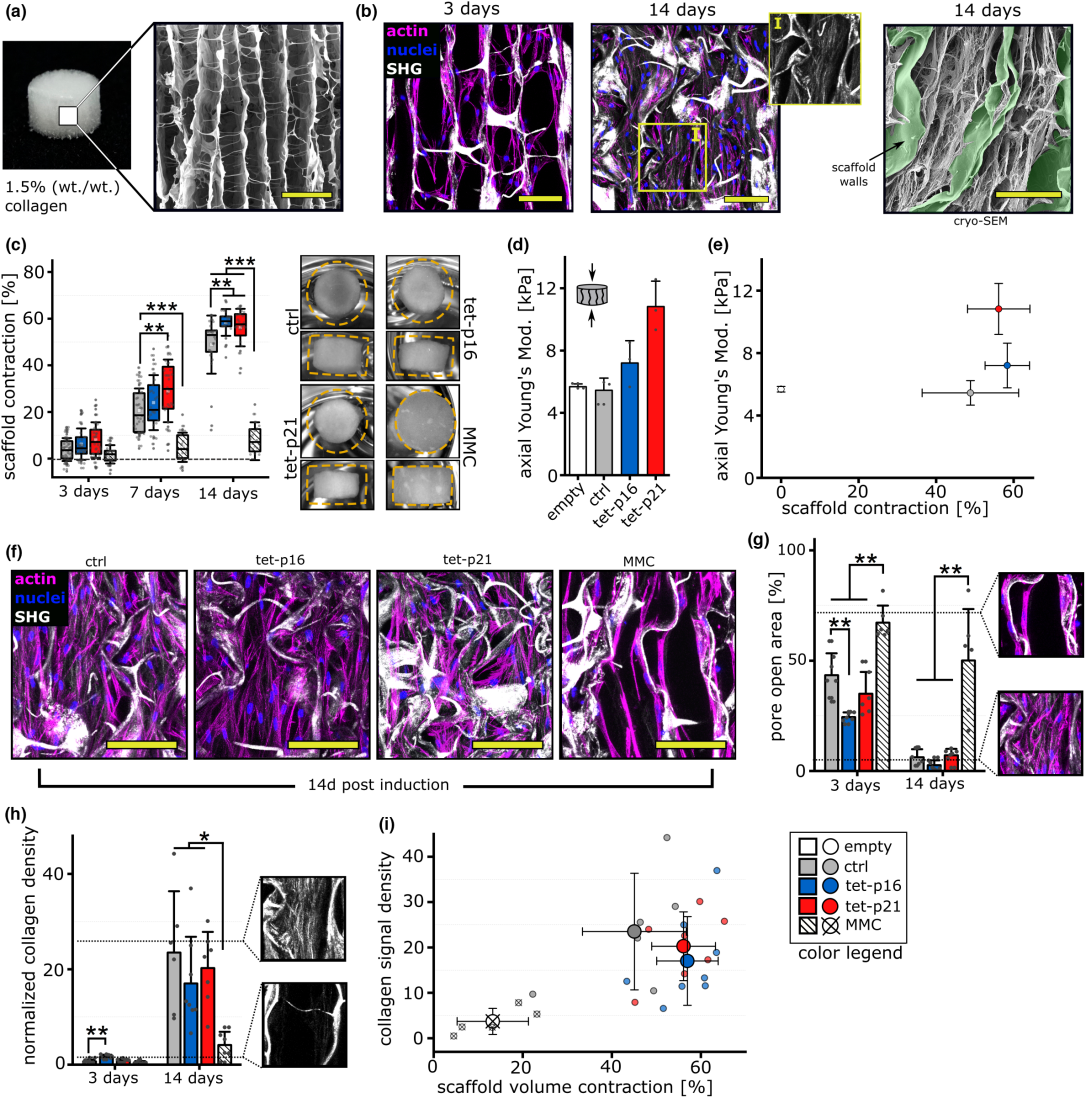
Modulation of macroscopic tissue formation and contraction through senescence. (a) Collagen scaffold (cylindrical sample shape) of 5 mm diameter and 3 mm height. Right: SEM shows the pore architecture. Scale bar 250 μm. (b) Confocal imaging 3 and 14 days post seeding of collagen scaffolds with primary human dermal fibroblasts. Samples were stained for Actin (magenta) and nuclei (blue). Fibrillar collagen was visualized by second harmonic generation (white). (I) Close‐up of ROI for SHG signal only. Cell derived fibrillar collagen is visible in between scaffold walls. Right: Cryo‐SEM after 14 days of culture. Scale bar 100 μm. (c) Macroscopic scaffold contraction (volume) relative to day 0. *N* > 20 (d) axial elastic modulus after 14 days of culture. (*N* = 3) (e) correlation of mean volume contraction and stiffness after 14 days of culture. (f) Confocal imaging after 14 days of culture. Samples were stained for Actin (magenta), nuclei (blue), and collagen was visualized by second harmonic generation (white). Scale bar 100 μm. (g) Pore open area after 3 and 14 days of culture. Close‐up images indicate partly (top) and fully closed (bottom) pores. *N* = 5–6 (h) Fibrillar collagen density normalized to the mean of 3 days control samples. Close‐up images indicate high (top) and low (bottom) collagen density inside pores. *N* = 5–6. (i) Correlation of scaffold contraction and collagen density after 14 days of culture. Small dots indicate single samples, large dots with errors represent means for each group.

We investigated the collective tissue‐forming capacity by culturing fibroblasts inside a macroporous collagen scaffold that serves as an in vitro wound healing model system (Brauer et al., 2019). The model allows to study aspects of tissue formation including cell organization, ECM formation, and the development of macroscopic mechanical tension that is part of the healing process. The biomaterial used in this model featured an elastic and reversible deformation under mechanical load together with a very high porosity of 98.5%. The open pore architecture provides space for cellular spreading, proliferation, and ECM deposition inside the pores (pore diameter D_Ø_ = 88 ± 21 μm, Figure 4a). The macroscopic compressive stiffness was quantified to be *E*_*axial*_ = 6 kPa along and *E*_*radial*_ = 1.3 kPa perpendicular to the cylinder axis, which is also the primary direction of the channel‐like pores. Subsequent to seeding into the collagen scaffolds, fibroblasts adhesion to and spread on on the thin collagen walls (Figure 4b, 3 days) followed by successive pore closure through the establishment of a dense cell‐ECM network with visible, cell‐derived fibrillar collagen bundles (Figure 4b, 14 days). Furthermore, cryo‐SEM indicated that in the course of tissue formation cells are mostly embedded in their own‐produced ECM reducing the contact with the scaffold material itself (Figure 4b, right image).

Subsequent to seeding of fibroblasts into macroporous collagen scaffolds at high cell densities, senescence was induced. We verified that induction efficacy was comparable to the 2D culture (Figure S4a–d). First, we monitored the scaffold volume contraction due to cell and ECM tensional forces building up within the scaffold pores for 14 days. At this point in time, tissue formation was shown to reach an equilibrium in the scaffold (Könnig et al., 2017) (Figure 4c). While control, tet‐p16 and tet‐p21 groups exhibited a mild degree of contraction at Day 3, contraction was completely absent in the MMC‐treated group. Even at Day 14 MMC‐treated cells showed only sparse contraction while all other groups contracted progressively. Intriguingly, an increased contraction was associated with p21 over‐expression already at day 7 while at Day 14 a significantly increased contraction was found for both p16 and p21. To exclude superimposed treatment‐specific effects, we verified that total cell counts did not vary significantly between groups. As expected, differences in cell density matched with differences in contraction (Figure S4e,f). The fact that the total cell number in the scaffold did not increase significantly from Day 3 to Day 14, even in the control group, can be attributed to the high cell density used that potentially induces a quiescent state of the cells within the material as a result of contact inhibition (Pavel et al., 2018). Since contraction is a result of ECM formation that leads to tissue densification and tensioning, we quantified the changes in axial stiffness after 14 days of culture (Figure 4d). While the axial stiffness of control group samples was comparable to the empty scaffold, tet‐p16 and p21 exhibited a progressive stiffening. This increase in stiffness at prolonged culture might explain why the increase in contraction particularly for tet‐p21 compared with control was rather large at Day 7 (50% difference between medians) and less pronounced at Day 14 (8% difference). The higher the stiffness of the tissue is, the more force (principle stress) is needed for further contraction. Thus, the system gradually approaches a limit where the tensional force generated by the cells and tensioned ECM fibers is counterbalanced by the increasing compressive force of the tissue. The endpoint of contraction thus represents an intrinsic endpoint with a non‐linear stiffness dependency (Figure 4e). Consequently, the apparent subtle differences in contraction in fact reflect much stronger differences in tissue‐internal tension.

As we have shown previously that tissue contraction is associated with progressive collagen fiber deposition, we performed confocal and second harmonic generation imaging (SHI) to visualize cell/tissue densification and fibrillar collagen deposition at Days 3 and 14 (Figure 4f). We first quantified the open pore area over time (Figure 4g). Fibroblasts bridge pores and dissect these into smaller segments which are subsequently closed by contraction, leading to a closing of voids and thus resembles an essential component of the wound healing process (Bao et al., 2020). Intriguingly, we observed that tet‐p16 but not tet‐p21 cells exhibit a significantly enhanced pore closure at Day 3, while MMC‐treated cells were not able to fill the voids at any time point. SHI revealed only little fiber deposition after 3 days, but already an increased density for tet‐p16 samples compared with control (Figure 4h). In line with the open pore area, this suggests that p16‐over‐expression might initially facilitate ECM deposition and filling of voids. This trend was not stable over time, as collagen density was at comparable levels for control, tet‐p16 and tet‐p21 groups at Day 14 (Figure 4h, Figure S4g). However, MMC treatment resulted in a significantly reduced collagen density at Day 14 which correlated well with our initial contraction analysis.

We recently observed for non‐senescent cells that macroscopic contraction and collagen density correlate linearly indicating a critical role of collagen fibers in building up internal tension (Brauer et al., 2019). Here, we noticed that, at comparable collagen signal density, scaffold contraction was enhanced for tet‐p16 and tet‐p21 compared to control cells, which further indicates and underlines the modulation of tissue formation due to cellular senescence (Figure 4i). As cellular senescence led to increased single cell forces, cells might be enabled to transfer larger force increments into tensioned collagen fibers and thus would require less collagen for a given degree of contraction. Since contraction leads to a densification of ECM, the amount of collagen formed under the different treatments was difficult to compare. We thus quantified the collagen density in a stiffer version of the scaffold (E_axial_ = 34 kPa) with the goal to monitor the collagen fiber deposition rate independent of contraction. While contraction was mostly absent for all groups, we observed a strong decline in collagen density for tet‐p16 and tet‐p21, which was even more pronounced for the MMC‐treated groups (Figure S4h,i). This emphasizes the generally reduced collagen deposition rate for senescent cells together with a more efficient usage of collagen fibers in tissue tensioning for tet‐p16 and tet‐p21 groups.

Together these data demonstrate that senescent cells actively engage in tissue formation, contraction, and tensioning. However, while tet‐p16 or tet‐p21‐induced (NDI) senescence entails distinct beneficial effects, MMC‐induced (DI) senescence resulted in a strong delay of tissue formation and tensioning—despite significantly increased single cell forces (Figure 3).

### 2.5 Antagonistic expression of ECM proteins & ECM remodeling enzymes

**FIGURE 5:**
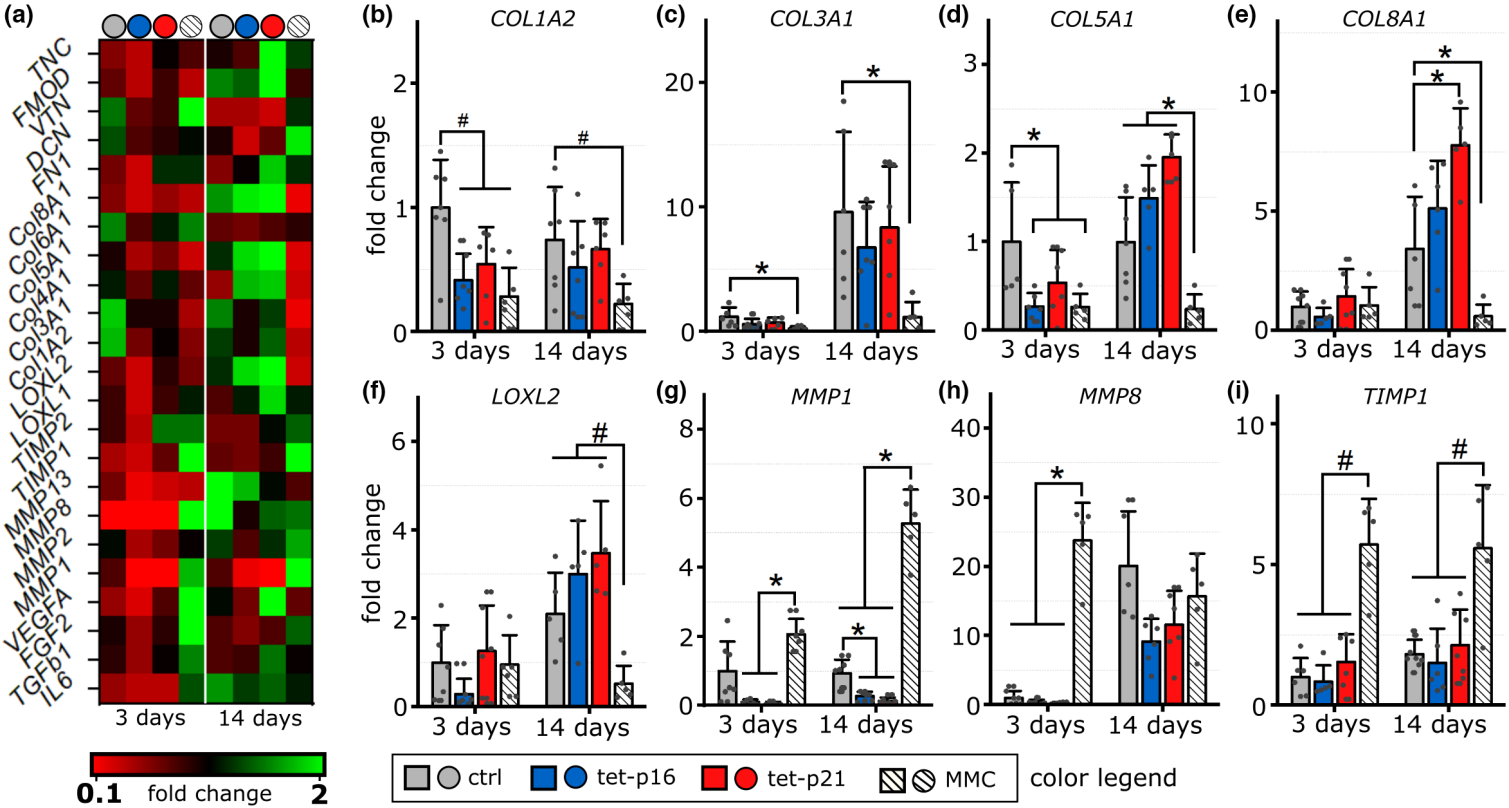
Regulation of gene expression in 3D through cellular senescence. (a) Heat map indicating the variance in between treatment groups and time points as a color code. (b–i) Gene expression analysis of collagen type I A2, type III A1, type V A1, type VIII A1, lysyl oxidase like 2, matrix metalloproteinase 1 and 8 and tissue inhibitor of metalloproteinases 1 3 and 14 days after induction of cellular senescence. Data are presented as fold change relative to the mean of 3 days timepoint of unstimulated (ctrl) cells. *N* = 5–6

Since collagen fibrillogenesis constitutes a key role in macroscopic tissue tensioning, we expected that expression patterns of collagens, collagen‐crosslinking or collagen‐degrading proteins were altered under senescence. Matrix metalloproteinases (MMPs) and tissue inhibitors of matrix metalloproteinases (TIMPs) are part of the SASP (Coppé et al., 2010) but also cytokines that potentially regulate the expression of collagens and glycoproteins. A comprehensive summary of regulations of ECM‐related proteins has been published recently, but while senescence generally seems to upregulate the expression of matrix degrading enzymes, collagens tend to be downregulated with senescence (Mavrogonatou et al., 2019). We performed gene expression analysis on glyco‐proteins, collagens, collagen‐remodeling, crosslinking enzymes, and growth factors relevant for tissue regeneration, 3 and 14 days after induction of cellular senescence (Figure 5a). Amongst fibril forming collagens, we observed only type I and III to be downregulated for all senescence treatments compared with control. Notably, MMC‐treatment showed the most pronounced reduction in expression (Figure 5b,c). Intriguingly, type V collagen, another fibrillar collagen was down‐regulated for all treatment groups 3 days after induction but showed slightly elevated expression for tet‐p16, tet‐p21 after 14 days compared to control. The same pattern was observed for network forming type IV and type VIII (Figure 5d, Figure S5a). Together this indicates that p16 and p21 over‐expression results in a shift in collagen expression patterns. In contrast to that, MMC treatment results in a general and consistent down‐regulation of various types of collagens.

Aside of collagens, the ECM consists of highly glycosylated proteoglycans (PGs) and fibrillar and non‐fibrillar glycoproteins. In particular, glycosylation of proteoglycans decreases with aging or in premature aging diseases (Chan et al., 2018; Li et al., 2013). We observed only little regulations of mRNAs for selected PG core proteins, but a significant reduction in the sulfated glycosaminoglycan content for MMC‐treated samples (Figure S5c–e). Selected glycoproteins, including fibronectin and tenascin‐c showed a high variance with a general trend for an increased expression with p21 over‐expression and slight down‐regulation with p16 over‐expression (Figure S5f,g). The expression of vitronectin, another glycoprotein was reduced for tet‐p16 and tet‐p21 but increased for MMC‐treated samples (Figure S5h). Generally, p21 over‐expression resulted in a more pronounced increase in expression compared to p16 for all collagens, PGs, and glycoproteins.

Extracellular collagen assembly is controlled by lysyl oxidases which mediate their crosslinking and collagen degrading MMPs. For the lysyl oxidase homolog 2 (LOXL2), we observed a slightly enhanced expression after 14 days upon p16/p21 over‐expression but a clear reduction through MMC‐treatment (Figure 5f). An almost identical expression pattern as for collagen type V and VIII (Figure 5d,e). Intriguingly, MMPs revealed an inverse expression pattern. MMP1 expression was consistently high upon MMC treatment for both time points, while p16 and p21 over‐expression reduced the expression compared to control (Figure 5g). Also, other MMPs behaved similarly, for example, MMP8 being strongly up‐regulated after 3 days (Figure 5h). Although described as part of the SASP, we did not find an enhanced expression of MMP13/collagenase 3 for any treatment group compared to controls (Figure S5i–l). Additionally, we observed a consistent upregulation of TIMP1 for MMC‐treated samples both at Days 3 and 14 (Figure 5i).

Besides ECM molecules, we analyzed distinct growth factors related to regeneration, including FGF‐2, IL‐6, TGF‐β1, and VEGFA (Figure S5m–p). Particularly, for FGF‐2 and IL‐6, MMC‐stimulation led to an increased expression after 3 days and partially even after 14 days, while p16 and p21 over‐expression provoked no major responses. As IL‐6 is a key factor of the SASP, these observations match with previous reports indicating that conditional over‐expression of p16 or p21 does not induce an SASP (Coppé et al., 2011).

Taken together, these data showed clear differences in the expression patterns between DI and NDI senescence. Furthermore, certain differences between tet‐p16 and tet‐p21 NDI senescence groups were visible, even though they shared a similar trend. In total, we demonstrated how cellular senescence affects cellular morphology and single cell mechanics and rendered consequences for macroscopic tissue formation processes in which senescent cells actively engage beyond the role of their paracrine signature.

### 2.6 Cell mechanics and ECM remodeling determine tissue contraction

As our investigations revealed that the senescence program modulates aspects of cell mechanics, collagen expression, and collagen remodeling/crosslinking, we next aimed at dissecting their relative contributions using inhibitors against individual cellular processes.

**FIGURE 6:**
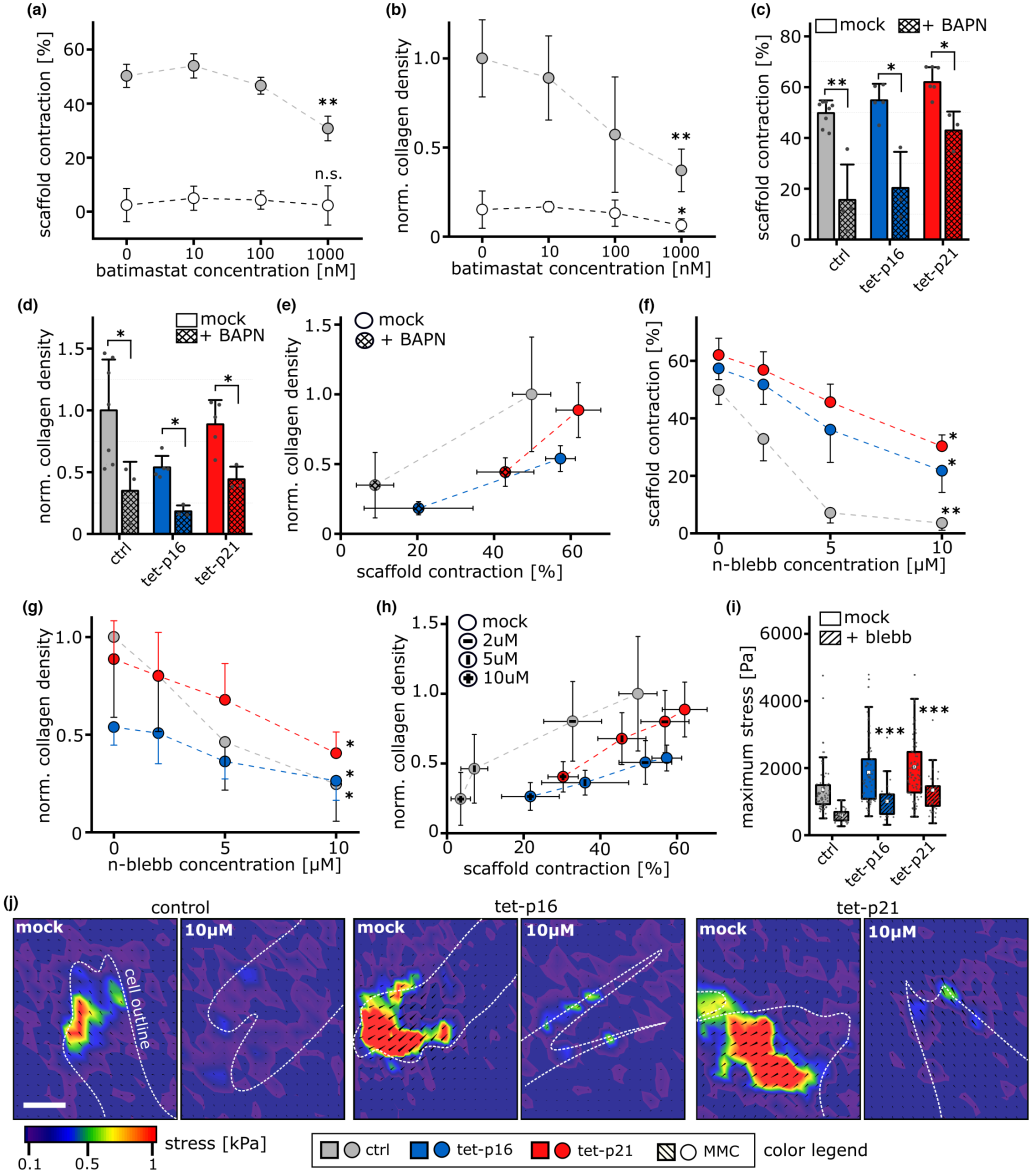
Macroscopic contraction depends on collagen crosslinking and cell mechanics (a) quantification of scaffold volume contraction after 14 days under treatment with Batimastat. (*N* = 3–4), significance level relative to 0 nM of each group. (b) Normalized fibrillar collagen density after 14 days under treatment with Batimastat. (*N* = 3–4), significance level relative to 0 nM of each group. (c) Scaffold volume contraction after 14 days under treatment with BAPN (β‐aminopropionitrile) (*N* = 4) (d) normalized fibrillar collagen density after 14 days under treatment with BAPN. (*N* = 4) (e) correlation of collagen density and volume contraction after 14 days under BAPN treatment. (f) Scaffold volume contraction after 14 days under treatment with nitro‐blebbistatin (n‐blebb). (*N* = 3–4), significance level relative to 0 nM of each group. (g) Normalized fibrillar collagen density after 14 days under treatment with n‐blebb. (*N* = 3–4), significance level relative to 0 nM of each group. (h) Correlation of collagen density and volume contraction after 14 days under n‐blebb treatment. (i) Maximum stress (Pa) detected for single cells either untreated (mock) or with 10 μM n‐blebb for at least 2 h. (*N* > 50). Significance level relative to control +10 μM blebbistatin. (j) Representative heatmaps of stress magnitude with overlaid displacement vectors (black arrows) for cell contact points. White dashed lines indicate cell outline. Scale 10 μm.

We used the broad MMP inhibitor Batimastat (BB‐94) to differentiate the effects of an increase in MMP and a reduction in collagen transcript levels after MMC‐treatment. The potent inhibition of MMP activity was verified in a zymographic assay (gelatin‐based for MMP2/9 activity) (Figure S6a). However, we did not observe significant differences, both in scaffold contraction and collagen formation after the application of Batimastat—except for very high (i.e., 10–100fold of the inhibitors' IC_50_) concentrations that reduced collagen density and scaffold contraction (Figure 6a,b). This observation suggests a negligible relevance of differences in MMP expression in our model system.

Aside of MMP‐mediated collagen degradation, lysyl oxidase‐dependent crosslinking might affect the ability to generate load‐bearing collagen fibers (Figure 5). We therefore monitored collagen deposition and contraction in the presence of β‐aminopropionitrile (BAPN), a known lysyl oxidase inhibitor (Canelón & Wallace, 2016), for ctrl, tet‐p16 and tet‐p21 samples (Figure 6c,d). We observed a reduction of contraction which was more pronounced for ctrl and tet‐p16 and less for tet‐p21 samples. Notably, the density of collagen was reduced upon BAPN‐treatment to a comparable extent for all groups. Compared with untreated control cells, tet‐p21 cells treated with BAPN generated a comparable degree of scaffold contraction after 14 days with less than 50% collagen density (Figure 6e). Tet‐p16 cells showed a similar trend. These results and the strong down‐regulation of lysyl oxidase upon MMC underline the prominent role of these enzymes in collagen fibril formation and tissue tensioning.

The observation of different degrees of contraction at comparable levels of collagen points to a high relevance of cell mechanics. Hence, we manipulated cellular forces by the application of nitro‐blebbistatin, a non‐cytotoxic blebbistatin derivative, during tissue formation (Képiró et al., 2014). We monitored scaffold contraction as a function of nitro‐blebbistatin concentration after 14 days for ctrl, tet‐p16, and tet‐p21 samples (Figure 6f). All groups showed a progressive decrease in contraction with increasing n‐blebb concentration. Intriguingly, at lower concentrations this decline was more pronounced for control samples. While the reduction in contraction at 5 μM relative to mock‐treatment was −86% for control samples, it was only −37% for tet‐p16 and −27% for tet‐p21. Hence, both in absolute and relative values, the tissue contraction capacity of p16 and p21 over‐expressing cells were less affected by the inhibitor. We further observed a dose‐dependent decline of collagen density in response to blebbistatin treatment, which was the expected result of reduced contraction. These observations illustrate that under mild cell force inhibition tet‐p16 and tet‐p21 cells achieve a comparable degree of contraction as control cells without inhibition (Figure 6h), thus highlighting the dominant influence of cellular forces for macroscopic tissue tensioning.

To exclude any group‐specific differences in the n‐blebb response, we quantified single cell forces under n‐blebb stimulation by TFM (Figure 6i,j, Figure S6b). All groups revealed a reduction in total force magnitude and particularly in maximum stress. Yet, we observed residual deformation fields for tet‐p16 and tet‐p21 but not for control cells illustrating that the relative differences in single cell mechanics were preserved between the groups. Considering the non‐linear stiffening of the 3D samples, this suggests that traction forces of control cells are reduced below a critical minimum level needed for scaffold contraction already at lower inhibitor concentrations compared to tet‐p16 and tet‐p21 cells, which might explain their faster and more sensitive reaction to increasing blebbistatin concentrations.

In sum, these data illustrate the combined influence of an altered cell mechanics and of collagen fibril formation on macroscopic tissue formation and contraction. While below a critical minimum collagen fibril deposition capacity (MMC, e.g., due to lack of collagen expression and crosslinking) tissue formation is generally ablated, above such a threshold partial compensation effects seem to occur in which a reduced collagen deposition (tet‐p16/p21) is counteracted by increased cell mechanics and collagen crosslinking.

## 3 DISCUSSION

**FIGURE 7:**
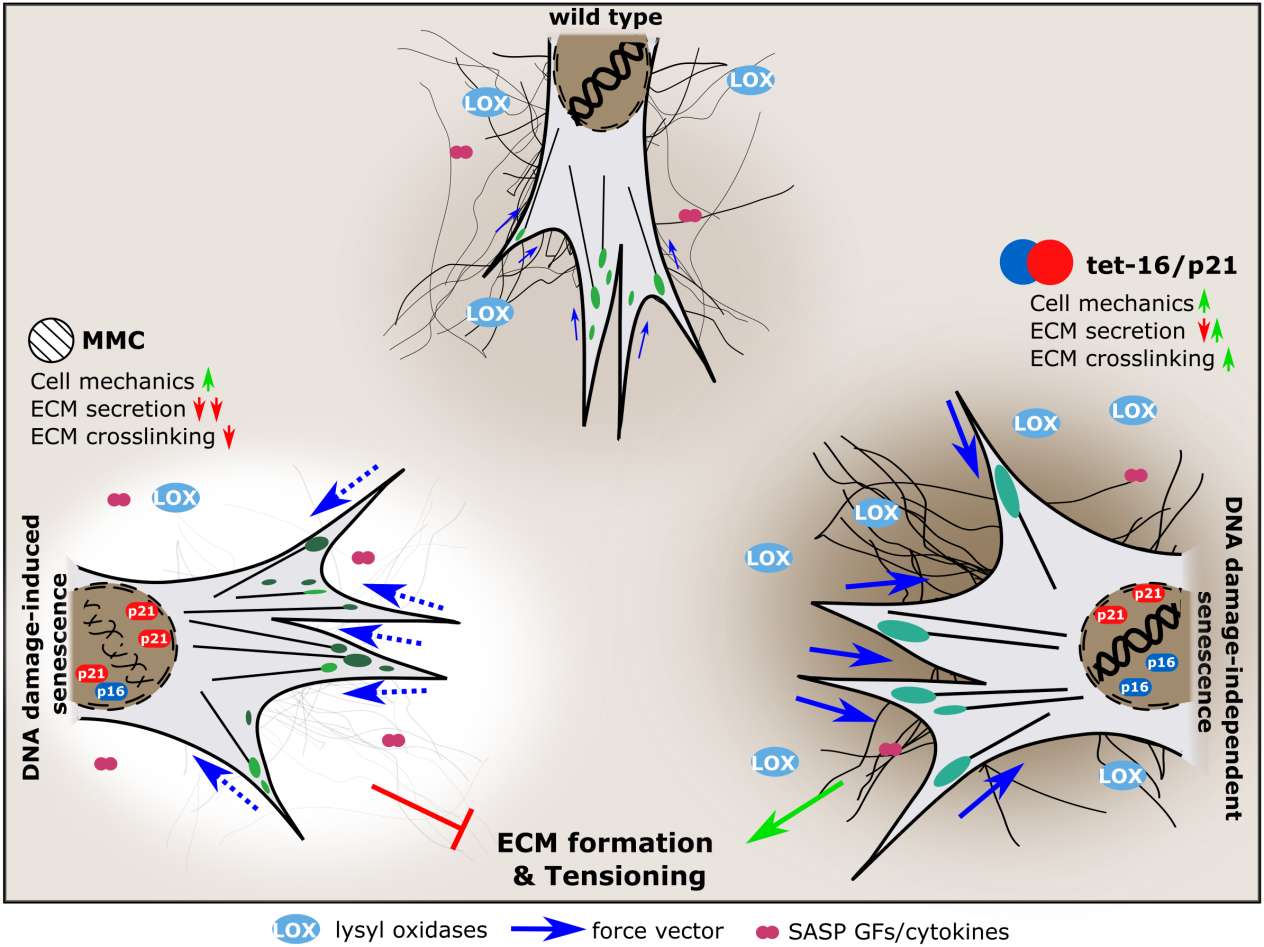
Regulation of tissue formation & tensioning by cellular senescence. Fibroblasts invade damaged tissue, exert mechanical forces onto the substrate and secrete growth factors, ECM, and ECM crosslinking molecules. Senescence increases single cell forces, but while DNA damage‐mediated senescence prevents tissue formation due to reduced collagen and LOX epxression, DNA damage‐independent senescence promotes pore closure and macroscopic tissue contraction. Both conditions potentially hold implications for various physiological and/or pathological scenarios such as embryonic development and tissue regeneration as well as age‐related tissue remodeling and cancer.

In contrast to a generally anticipated detrimental role of senescence during aging, increasing evidence suggests that the transient presence of senescent cells entails distinct beneficial features in the context of embryonic development and tissue regeneration (Da Silva‐Álvarez et al., 2020; Demaria et al., 2014; Muñoz‐Espín et al., 2013; Ritschka et al., 2017). On the contrary, the occurrence of p21‐positive cells in wounds of aged individuals was described to correlate with a delay of healing (Chia et al., 2021; Jiang et al., 2020). So far, the effects of senescent cells were primarily attributed to paracrine signaling as their secretory signature comprises cytokines such as IL‐6, PDGF‐A, and VEGF but also MMPs counteracting fibrosis (Krizhanovsky et al., 2008). In this study, we describe cellular changes induced by senescence that might point towards a mechanical role of senescent cells during tissue formation beyond the role of soluble SASP factors as summarized in Figure 7.

We compared classical DNA damage‐induced (DI) senescence (MMC treatment), with a DNA damage‐independent persistent growth arrest through conditional over‐expression either of p16 or p21 (NDI senescence). Our data show that MMC‐treated cells mainly over‐express p21, which makes them best comparable to tet‐p21 cells. However, the level of DNA damage was strongly enhanced in DI, whereas in tet‐p21 cells seemed even lower than in control cells. Thus, genomic integrity and cellular “health,” might be higher in these cells, although they show indicators of senescence. It hereby underlines the necessity to apply multiple‐markers for the determination of senescent cells and their subsets (Gorgoulis et al., 2019; Saul et al., 2022). A detailed evaluation of our DI and NDI senescence models revealed that around 40% of MMC‐treated cells were distinct from controls based on nuclear pH2A.X signal. The percentage of tet‐p21 cells expressing p21 was in a similar range, while more than 50% of tet‐p16 cells showed p16 expression. However, bulk protein levels demonstrated a roughly 5‐fold elevation of p16 and p21 levels in their respective cell lines while p21 expression was increased around 2‐fold in MMC‐treated cells. These results demonstrate sufficient physiological properties of our cellular models. A higher proportion of p16‐ or p21‐expressing cells might have been obtained by clonal selection, which, however, would have required many more cell divisions potentially leading to the induction of replicative stress and senescence. Clonal cells are not necessarily representative for the whole cell population. We therefore opted for a positive selection strategy, which is never 100% selective due to protection through bystander cells. Although representing a physiologically occurring situation, the expression heterogeneity can be regarded as a limitation in terms of that the observed effects cannot be correlated with the expression strength of individual cells or homogeneous populations. Yet, such a limitation similarly exists for most DNA damage‐mediated model systems (including MMC treatment) as marker‐based sorting would result in populations of few cells which cannot be expanded. Furthermore, the percentage of transgenic cells might in fact be higher than 40%–50% due to limited transactivator levels that might, for example, be triggered by its spontaneous epigenetic silencing (Duran et al., 2022).

Although being regulated in their expression, MMPs exhibited little relevance in the general modulation of collagen formation and tensioning in our 3D tissue model as inhibition did not lead to a recovery of the reduced collagen formation observed with induction of senescence (Figure 6a,b). This might be explained by the fact that our investigations focus on tissue formation and repair and not on homeostasis and matrix remodeling where the relevance of MMPs is more pronounced (Jun et al., 2020).Our data confirm that even mechanically competent cells require load‐bearing collagen fibers for the generation of macroscopic tension via the previously described slip‐and‐ratchet mode of tissue contraction (compare MMC and p16/p21 groups in Figure 3c and 4i) (Brauer et al., 2019). This is particularly relevant for the tissue healing cascade beyond the hematoma phase in which the very soft environment (2–4 kPa) can be contracted by cell force alone. Although not being the primary focus of this work, we found indications for a stiffness‐dependent regulation of myo‐fibroblast activation by DI‐senescence (Figure S3) which might modulate cell contractility in the course of tissue formation. The strong relevance of cell mechanics is underlined by our inhibitor studies which indicate that the apparent contraction detected after 14 days does not reflect strong differences in cell mechanics and tissue stiffness (Figure 6). The more contraction progresses, the stiffer the samples become, and the more force needs to be applied to realize further contraction. Hence, an endpoint in contraction correlates with a peak cell force. Since the dependency of contraction and stiffness is non‐linear (Figure 4e), a lower maximum force (Fmax) of control cells makes their 3D micro‐tissue constructs react with a stronger reduction in contraction already to lower concentrations of blebbistatin. On the contrary contraction of the stiffened tissue (p16 and p21) requires an over‐proportional increase in cell force. Consequently, the rather mild increase of scaffold contraction observed for p16, p21 vs. control (Figure 4c) after 14 days requires a significant increase in internal tissue tension (Figure 3c–f) for which a critical minimum density of tension bearing collagen fibers is required (MMC vs. p16/p21 in Figure 4i). Intriguingly, differences between p16 and p21 overexpressing cells were rather subtle but consistently present and alterations of gene expression compared to control cells were more pronounced for tet‐p21 in various aspects (collagens & LOXL), cell mechanics (Fmag) and inhibitor response (n‐blebb). Up to now, however, we cannot exclude that differences are a result of slightly different expression profiles and population distributions (e.g. percentile amount of marker‐positive cells).

Beyond soluble factors, the secretome comprises insoluble ECM components that are locally more confined and persistent that further store and present growth factors. As such, the ECM has the potential to instruct other cells even when the ECM‐forming cell is no longer present. Matrix deposited by senescent cells influences the proliferation of naïve cells (Hiebert et al., 2018). Similar observations were made with aged tissues (Ozcebe et al., 2021). Whether cells directly react to the matrisome signature or whether an age‐associated loss of glycosylation (Chan et al., 2018) indirectly affects matrix‐guided growth factor signaling remains elusive so far. Our data confirm previous reports of alterations in ECM composition in aging (Mavrogonatou et al., 2019), specifically indicating a general downregulation of collagen type I for all treatment groups (MMC, p16, p21). Yet, while also other collagens (type III, V, VIII) were downregulated under MMC‐treatment, tet‐p16 and p21 over‐expressing cells revealed an increased expression of these additional fibrillar and network forming collagens (Figure 5). Intriguingly, the strong down‐regulation of MMPs with tet‐p16/p21 overexpression does not match with other reports (Vamvakas et al., 2017) where nucleus pulposus cells were used. This might point to a tissue/cell type‐specific response beyond the question of DI vs. NDI senescence.

In aggregate, this study sheds light on how individual aspects of the cellular senescence program influence tissue formation and tensioning. It underlines the active role these cells exert in the process of tissue formation in addition to the canonical paracrine signaling. The data provided here further highlight the ambivalent nature of the senescence phenotype which is capable to influence fundamental physiological and pathological processes even independent of the aging process. While selective cell cycle arrest results in enhanced cell contractility and ECM crosslinking, the lack of ECM secretion that additionally concurs with DNA damage‐mediated senescence oppresses such a potentially beneficial role as these cells lack the essential ECM elements in order to transfer and store cell forces. In respect to tissue regeneration, this motivates a closer look at individual aspects of senescence and their respective role in the healing process. The contraction of wounds and tissue defects are an essential process in healing. The alternative strategy of senescent cells to achieve contraction through a less dense network of collagen‐I fibers but increased cell forces, might be of relevance in avoiding scar tissue formation. In the future, these effects might be therapeutically exploited by enhancing beneficial and suppressing detrimental aspects of cellular senescence.

## 4 EXPERIMENTAL PROCEDURES

### 4.1 Cloning of transposon constructs

For Tet‐inducible overexpression of p16^INK4a^ and p21^Cip1^ in human primary fibroblasts we used a two‐component transposon system (Figure S1). First, the puromycin resistance gene with a preceding 2A site was integrated into the transposable region of the tet‐controllable transposon plasmid pTOV‐11. This DNA fragment was amplified from the vector pSpCas9n(BB)‐ 2A‐Puro (PX462) V2.0 (Addgene #62987). This was followed by integration of the sequence into the target vector via restriction digestion by BamHI and Crf9I. Integration of the p16^INK4a^ or p21^CIP1^ cDNA into the target vector followed in another cloning step. The cDNA was amplified from vectors pBabepuro3‐p16Flag (Addgene #24934) and Flag p21 WT (Addgene #16240) and integrated into the transposon vector via NotI and SalI restriction sites.

### 4.2 Cell isolation and culture

Primary human dermal fibroblasts were isolated from human skin biopsies via outgrowth culture. Collection of human skin samples was approved by the Institutional Review Board of the Charité Berlin as well as the patient's written consent. Fibroblasts were cultured in Dulbecco's Modified Eagle Medium (DMEM, 11960‐044; Thermo Fischer) supplemented with fetal bovine serum (FBS 10% (v/v), S0115; Biochrom AG), Penicillin (100 U/ml) and Streptomycin (100 μg/ml) (1% (v/v) A 2213; Biochrom AG), and nonessential amino acids (NEA, 1% (v/v), K0293; Biochrom AG) at 37°C with 5% CO2 in a humidified incubator.

### 4.3 Transfection and antibiotic selection

Fibroblasts with stable transposon integration were generated by transfection of the abovementioned transposon constructs with a SB100X sleeping beauty transposase expression vector pCMV(CAT)T7‐SB100(AL) into primary human fibroblasts using the Neon® Transfection System (990 V, 40 ms) (Thermo Fischer) (Mátés et al., 2009). Transposon‐positive cells were selected by addition of 1 μg/ml puromycin (P9620, Sigma Aldrich) to the culture medium over 2 weeks.

### 4.4 Induction of cellular senescence

Cellular senescence was induced for genetically engineered cells (p16^INK4^ or p21^CIP^ over‐expression) by stimulation with 0.5 μg/ml doxycycline hyclate (D9891, Sigma Aldrich). Fresh doxycycline was repeatedly added every 2–3 days of culture or together with medium exchange after 7 days. DNA damage was induced by stimulation with 1 μg/ml mitomycin C for 24 h. Afterward, the medium was removed and replaced by fresh expansion medium.

### 4.5 Inhibitors

Batimastat was purchased from abcam (ab142087), (S)‐4′‐nitro‐Blebbistatin from Cayman Chemical (24171), β‐aminopropionitrile from Sigma Aldrich (A3134).

### 4.6 Flow cytometry

Flow cytometry analysis was performed on a BD FACSAriaII SORP (BD Biosciences, San Jose, CA, USA), configured with 4 lasers (violet, blue, yellow‐green, red) at the Charité | BIH Flow & Mass Cytometry Core Facility. Flow cytometry data analysis was performed with FCSExpress 7 Research.

### 4.7 Proliferation analysis

Fibroblast cells were plated at a density of 6600 cells/cm^2^ (=30% confluency). One plate was harvested after overnight adhesion and prior to induction of cellular senescence by washing once with PBS and freezing of the plate at −80°C. Plates were further harvested after 3, 7 and 14 days. Proliferation was quantified using the CyQUANT™ Cell Proliferation Assay (C7026, Thermo Fischer) according to manufacturer's instructions. The fluorescence signal was determined using a plate reader (Infinite PRO, Tecan) with excitation at 480 nm and emission at 520 nm. Raw fluorescence values were converted into cell count using a standard curve created from a cell pellet of a defined amount of cells and further normalized to day 0 time point to obtain values of population doublings.

### 4.8 β‐Galactosidase activity assay

The senescence state of fibroblast populations was determined using the Senescence β‐Galactosidase Staining Kit (#9860, Cell Signaling Technology) according to the manufacturer's instructions. In brief: fibroblasts were plated at 6600 cells/cm^2^ (=30% confluency) and kept in culture for 7 or 14 days. Subsequently, cells were rinsed with 1× PBS, fixed with 1× Fixative Solution, and rinsed twice with 1× PBS. Afterwards β‐Galactosidase Staining Solution was applied to the cells for overnight incubation at 37°C in a dry incubator (no CO_2_). The next day, images of cells were acquired using a conventional inverted microscope. Total cell count and β‐galactosidase positive cells were determined by manual counting.

### 4.9 Gene expression

RNA was isolated using the PureLink® RNA Mini Kit (12183018A; Thermo Fischer) in combination with PureLink® DNase (12185010; Thermo Fischer) to digest genomic DNA according to the manufacturer's instructions. RNA was reverse transcribed into cDNA using the iScript™ cDNA Synthesis Kit (170‐8891, Bio‐Rad) according to manufacturer's instructions. Real‐time PCR was performed with an iQ5™ Real‐Time PCR Detection System (Bio‐Rad) using iQ™ SYBR® Green Supermix (170‐8882, Bio‐Rad). Mean normalized expressions were calculated from C_T_‐values using the ΔC_T_‐method with correction for primer efficacy (Pfaffl, 2001; Ramakers et al., 2003). Data were finally presented as fold change. Primer sequences are summarized in Supplementary Table 1.

### 4.10 Immunoblotting

Cell lysates were prepared using RIPA buffer (9806, Cell Signaling) supplemented with protease and phosphatase inhibitors (11836153001 and 4906845001; Roche) according to manufacturer's instructions. Lysates were mixed with 4× Loading Buffer (928‐40004, Li‐Cor) and denatured at 95°C for 5 min prior to electrophoresis. SDS‐PAGE was performed with the NuPAGE® electrophoresis system (Thermo Fischer) including 4%–12% Bis‐Tris gels (Thermo Fischer, # NP0336BOX) and MES SDS Running Buffer (NP0002; Thermo Fisher) according to the manufacturer's instructions. Western blotting onto nitrocellulose membranes (10600002; GE Healthcare) was performed inside an XCell II™ Blot Module (Thermo Fischer) for 1 h at 30 V constant (25 mM Tris‐BASE; 192 mM Glycine, 20% (v/v) Methanol transfer buffer). Membranes were washed with TBS (15 mM Tris–HCl pH 7.6; 136 mM NaCl) and blocked with TBS Blocking Buffer (927‐50000, Li‐Cor). Primary antibodies were incubated at 4°C overnight according to manufacturer's instructions. The following antibodies were used: Phospho‐Histone H2A.X (Ser139) (2577, Cell Signaling), Cyclin D1 (2978, Cell Signaling), Lamin B1 (13435, Cell Signaling), p16^Ink4a^ (80772, Cell Signaling), p21^Cip1^ (2948, Cell Signaling), GAPDH (2118, Cell Signaling), FLAG (740001, Thermo Fischer), pMLC (3675, Cell Signaling), and alpha‐SMA (ab5694, Abcam).

### 4.11 Zympgraphy

Conditioned media were collected after 7 days of culture and concentrated to 5× using Amicon Ultra centrifugal filter units (10 kDa, UFC501096, Merck). Concentrates were loaded onto gelatin zymogram gels (ZY00100BOX, Thermo Fischer) and processed according to the manufacturer's instructions. Developed gels were stained using SimplyBlue Coomassie stain (LC6060, Thermo Fischer).

### 4.12 Scratch migration assay

Scratch assay was performed as described previously (Liang et al., 2007; Pumberger et al., 2016). In brief: 6 × 10^4^ (ctrl) or 5.5 × 10^4^ (senescent cells, all conditions to correct for increased cell area) fibroblast cells were seeded into a 24 well plate to obtain a confluent monolayer. After overnight adhesion, a scratch was created using a 200 μl tip. Cell layers were washed twice with PBS and incubated with migration medium (DMEM high glucose supplemented with P/S and NEA). Immediately after addition of migration medium, recording of the scratch area was started using an inverted microscope (DMI6000B, Leica). Images were taken at 60 min interval over a period of 24 h, and analysis was performed using TScratch (Gebäck et al., 2009).

### 4.13 PDMS substrate preparation

Soft polydimethylsiloxane substrates were prepared using Sylgard 184 (Dow Corning, MI, USA) in a 1:70 ratio of curing agent to base. The mixture was degassed and polymerized at 70°C for at least 2 h. Substrates were sterilized with 70% ethanol for at least 30 min and then washed with ultrapure water (UPW). Substrates were functionalized with 0.1 mg/ml dopamine (H8502, Sigma Aldrich) dissolved in 10 mM Tris, pH 8.5 overnight (Lee et al., 2007) and washed 2–3× with UPW. Substrates were finally coated with 1 μg/cm^2^ fibronectin (341635, Merck) in PBS for 30 min at 37°C. Substrates were washed once with PBS and directly used for cell seeding.

### 4.14 Mechanical testing

Axial compression testing was performed as described previously (Brauer et al., 2019).

### 4.15 3D culture

Macroporous collagen scaffolds (Matricel GmbH) were prepared from sheets using a biopsy punch to obtain cylindrical samples (5 mm Ø, 3 mm height). Scaffolds were immersed in a concentrated cell suspension (7500 cells/μl) and incubated for 1 h at 37°C in a humidified incubator without additional medium followed by addition of fibroblast growth medium (10% FBS) for 4 h. Afterward, doxycycline hyclate and mitomycin C were added to the culture to a final concentration of 0.5 and 1 μg/ml, respectively. Medium was replaced the next day by DMEM containing 2% FBS and 1.36 mM L‐ascorbic acid 2‐phosphate (#49752, Sigma‐Aldrich) to allow collagen fibrillogenesis. Concentrations of P/S and NEA are equal to cell expansion medium. As for 2D, fresh doxycycline was added every 2–3 days, and the medium was exchanged once after 7 days of culture.

### 4.16 Scaffold contraction analysis

Contraction analysis of macroporous collagen scaffolds was performed as described previously (Brauer et al., 2019). In brief: samples were scanned in both directions at the respective time points (0, 3, 7, 14 days) in cell culture medium using a digital scanner with a resolution of 1200dpi. The cylinder cross sectional area was determined by manual contouring of the sample outline (sample top view). The sample height was calculated based on the mean distance of top and bottom surface contour lines (sample side view) and samples volume was calculated by multiplying sample cross‐sectional area and height. Scaffold volume contraction is expressed as the percentile change relative to the sample volume at Day 0.

$$C_{\text{volume}}\left(\%\right)=\frac{(V_{0}-V_{t)}}{V_{0}}∙100$$

### 4.17 Histology

Samples were fixed using 4% paraformaldehyde solution. The fixation was quenched with 25 mM NH_4_Cl/PBS. In case of 3D samples, scaffolds were incubated in 5% gelatin, 5% sucrose dissolved in PBS at 37°C with subsequent solidification at 4°C to enable cutting of cylindrical samples into halves. Cylinder halves were embedded into Tissue‐Tek* O.C.T. Compound (#25608‐930, Sakura Inc.) and planed using a CryoStat (LEICA CM3050S). Samples were rinsed thoroughly in PBS at 37°C to remove gelatin/sucrose and Tissue‐Tek, respectively. The following antibodies were used: mouse‐anti‐vinculin (#V9131, Sigma Aldrich), FLAG (740001, Thermo Fischer) and goat‐anti‐mouse IgG‐488 (#A‐11029, Thermo Fischer). F‐actin and cell nuclei were visualized using SYTOX™ Green (#S7020, Thermo Fischer), Draq5 (#424101, Biolegend) and Phalloidin‐Atto550 (19083, Sigma Aldrich).

### 4.18 SEM

Fixed samples were incubated 2 × 15 min each in an ascending alcohol series of 50%, 70%, 80%, 96%, and 100%. Samples were dried in a critical point dryer (EM CPD300, Leica). Freeze‐dried samples were gold sputtered and imaged using the JCM‐600 (JEOL GmbH) device.

### 4.19 Imaging

Microscopy was performed on a Leica SP5 confocal microscope combined with a Mai Tai HP multiphoton laser (Spectra Physics). Imaging of focal adhesions was performed with a 63‐fold water immersion objective at a resolution of 0.12 μm × 0.12 μm × 0.99 μm voxel size. Collagen fibers together with actin and nuclei signals were recorded using a 25‐fold water immersion objective at a resolution of 0.6 μm × 0.6 μm × 4 μm voxel size. Fibrillar collagen was recorded by second harmonic generation (Chen et al., 2012) with constant laser power and detection parameters for all samples (910 nm excitation, 440–460 nm detection).

### 4.20 Image analysis

All image analysis steps were performed using Fiji.

#### 4.20.1 Focal adhesion analysis

For image analysis, a single plane was selected from each stack, which was close to the substrate surface. The image was processed by the consecutive application of subtract background, median and enhance contrast functions. A threshold was applied to select focal adhesions and objects were analyzed by the analyze particles function (size >0.073 μm^2^ as exclusion criteria for single pixel signals). Focal adhesions were classified according to their size into groups of tiny (<0.25 μm^2^), small (0.25–1 μm^2^), medium (1–5 μm^2^) and large (>5 μm^2^). The cell area and aspect ratio was obtained from manual contouring of cells.

#### 4.20.2 Collagen density

The pores of the scaffold were manually contoured to obtain the ROI for analysis. A stack of 8 slices (volume 620 μm × 620 μm × 28 μm) was converted into a sum projection (sum slices) and the total signal was obtained by summation of all pixels within the ROI. Collagen signal density was obtained by normalizing the sum signal to the ROI volume and further normalized to the mean of 3 days control samples. To ensure representation of the full sample, at least 5 spots were selected per sample at multiple positions to cover rim (top, bottom, left, right) and center.

#### 4.20.3 Cell density

A stack of 8 slices was converted into a maximum projection and the cell count was obtained by applying the analyze particles function. Finally, the cell count was normalized to the stack volume to obtain a value of cell density expressed as cells per mm^3^.

#### 4.20.4 Open pore area

The area of each pore was obtained from manual contouring of SHG signal and the open area from manual contouring of the respective actin channel.

### 4.21 Traction force microscopy

TFM was performed based on previously established protocols for polyacrylamide gel casting and coating (Plotnikov et al., 2014) and data analysis (Martiel et al., 2015; Tseng et al., 2012). In brief: Coverslips were activated with 50% (v/v) 3‐Aminopropyltrimethoxysilane (APTMS, #281778, Sigma Aldrich) followed by glutaraldehyde fixation using a 0.5% (v/v) solution (#G5882, Sigma‐Aldrich). Thin polyacrylamide gels (PAA) were prepared by a sandwiched PAA master mix in between activated cover‐slips and RainX‐treated microscope slides. The PAA master mix contained FluoSpheres (0.1 μm, #F8800, orange; 0.2 μm #F8805, blue) acrylamide (#161‐0140;Bio‐Rad), bis‐acrylamide (#161‐0142; Bio‐Rad) and ammonium persulfate solution (APS, # A3678; Sigma‐Aldrich) and tetramethylethylendiamin (TEMED, #161‐0800; Bio‐Rad) to induce polymerization. Gels were thoroughly rinsed with deionized water before coating with 5 μg/cm^2^ fibronectin (#341635, EMD Milipore) using UV‐activated (2 × 200 s 10 mW/cm^2^) sulfosuccinimidyl 6‐(4′‐azido‐2′‐nitrophenylamino)hexanoate (Sulfo‐ SANPAH, #22589, Thermo Fischer). Gels were again washed at least 4 times with PBS under sterile conditions and seeded with cells at a density of 3000 cells/cm^2^. Cells were stained using CellTracker Green CMFDA (#C7025, Thermo Fischer) prior to image acquisition. Therefore, slides were transferred into a custom‐made perfusable chamber which was mounted onto a Leica SP5 laser scanning microscope equipped with a 63 fold water immersion objective. Images were recorded with a spatial resolution of 0.24 μm × 0.24 μm × 0.25 μm. Cells were removed by trypsinization, and beads were recorded for marked cell positions to obtain image pairs. Total force magnitude and mean/maximum stress, respectively, were calculated using Fiji plugins for Particle Image Velocimetry and Fourier Transform Traction Cytometry with regard of the cell outline based on CTG signals (Tseng et al., 2012).

### 4.22 Statistics

All plots were created using the OriginPro 2020 (OriginLab Corporation) software. Data are presented as mean values with standard deviation. Individual data points are overlaid as dark grey dots. Boxplots are drawn as boxes with 25% lower and 75% upper limit. The mean value is shown as a white square and the median as black line. Statistical significance was assessed using a two‐sided Mann–Whitney‐*U* test. *p*‐values were adjusted by Bonferroni correction in case of multiple testing of groups. A *p*‐value of <0.05 was considered as statistically significant. Different significance levels are indicated as: #*p* < 0.1, *p < 0.05, **p < 0.01, ***p < 0.001.

## AUTHOR CONTRIBUTIONS

E.B., T.L., D.K., M.G., U.K., and A.P. designed the experiments. E.B., T.L., D.K., S.G., S.C., and J.K. carried out the experiments. E.B. and T.L. analyzed the experiments. E.B., U.K., and A.P. wrote the manuscript. All authors approved the final version.

## CONFLICT OF INTEREST

The authors declare no conflict of interest.

## Supporting information


Supinfo

## Data Availability

The data that support the findings of this study are available from the corresponding author upon reasonable request.
