# Supplementary material for: Dissecting the influence of cellular senescence on cell mechanics and extracellular matrix formation in vitro
Source: Aging Cell. 2022 Dec 13;22(3):e13744. doi: 10.1111/acel.13744 (PMC10014055; doi:10.1111/acel.13744)
Supplement: Supplementary file 1 — Supinfo [file ACEL-22-e13744-s001.docx]

**Supplement**

**
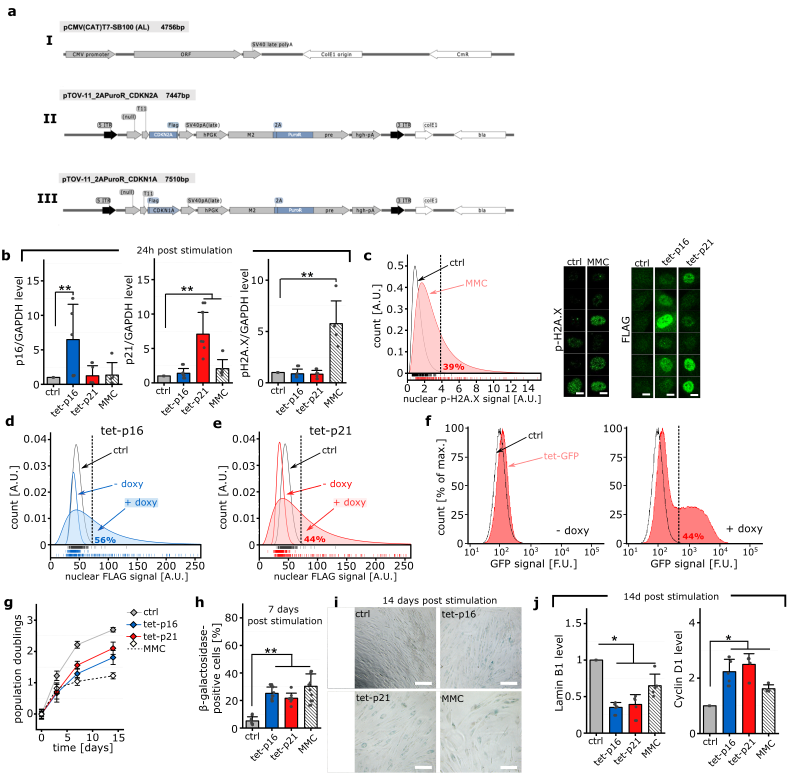
**

**Figure S1: (a)** Maps of the constructs used for stable transposon integration**.** (I) pCMV(CAT)T7-SB100 (AL) vector for expression of hyperactive sleeping beauty transposase (SB100X). (II) pTOV-11_2APuroR_CDKN2A vector containing the *CDKN2A* cDNA and a puromycin resistance flanked by LTRs of the sleeping beauty transposon. (III) pTOV-11_2APuroR_CDKN1A vector containing the *CDKN1A* cDNA and a puromycin resistance flanked by LTRs of the sleeping beauty transposon. **(b)** Quantification of western blot signals for p16, p21 and p-H2A.X (Ser139) 24 hours after stimulation. Signals are normalized to GAPDH levels as loading control and expressed as fold change relative to control samples (n=4-5). **(c)** Histogram showing nuclear signal of phospho H2A.X for control and MMC-treated cells. right: representative nuclei for p-H2A.X and FLAG. Scale bar: 10µm. (d) Histogram showing nuclear FLAG signal of tet-p16 cells compared to control. Dashed line indicates 95% quantile of control population. (e) Histogram showing nuclear FLAG signal of tet-p21 cells compared to control. Dashed line indicates 95% quantile of control population.**(f)** Histogram showing flow cytometry data for tet-GFP-transfected fibroblasts. Black line represents non transfected cells. **(g)** Proliferation curves expressed as fold change (logarithmic ln2) relative to day 0 (=population doublings) over a time frame of 14 days. Measured time points are presented as colored circles interconnected by lines. (n=9). **(h)** Percentile abundance of β-galactosidase-positive cells 7 days post induction of cellular senescence (n=9). **(i)** Representative images of SA-β-gal stainings 14 days post induction of cellular senescence. Scale bar 200µm. **(j)** Quantification of western blot signals for Lamin B1 and Cyclin D1 14 days after induction of senescence. Signals are normalized to GAPDH levels as loading control and expressed as fold change relative to control samples (n=4-5).


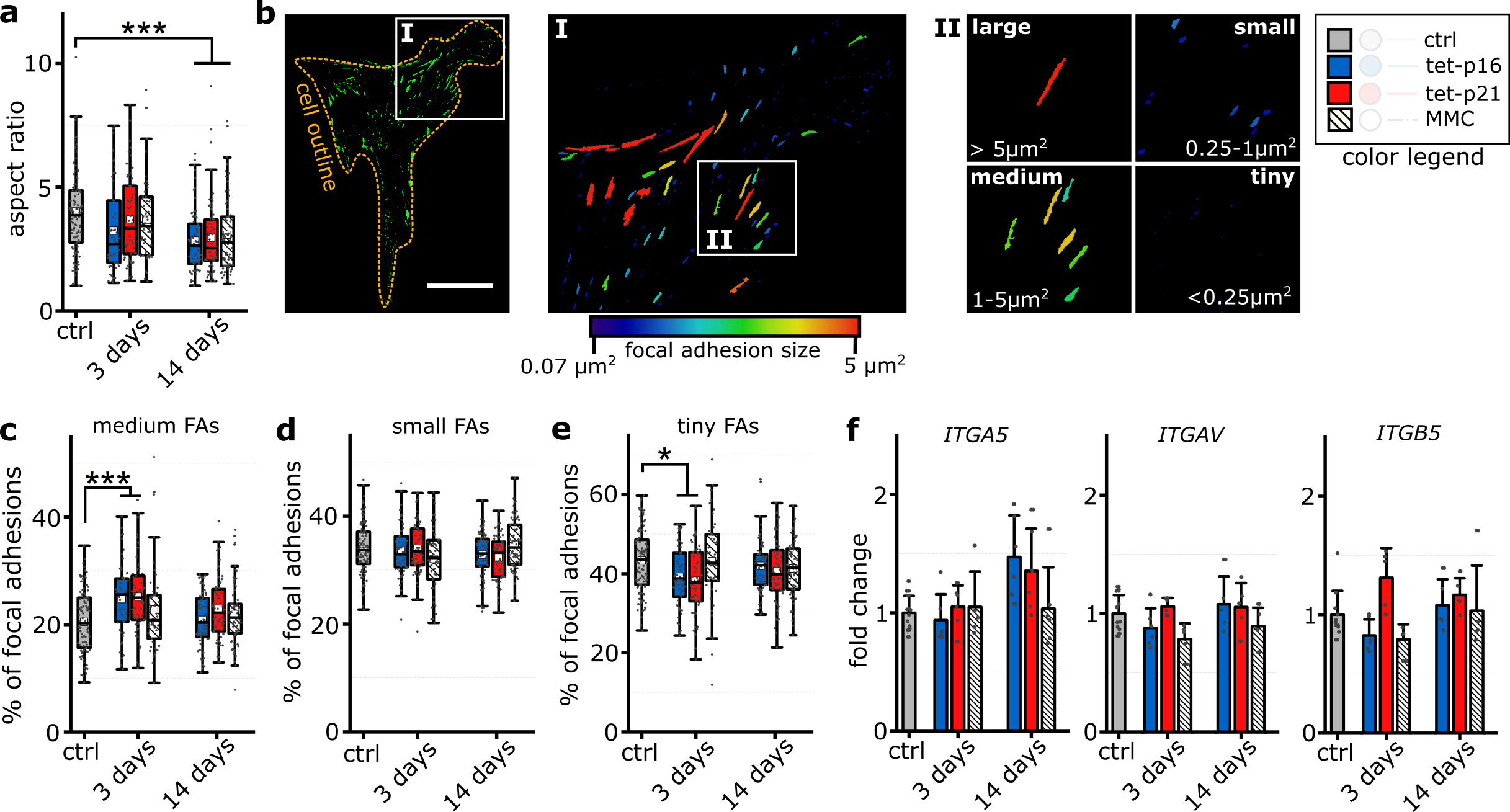


**Figure S2: (a)** Quantification of cellular aspect ratio (n≥50). **(b)** Confocal image of vinculin staining (green) with cell contour outlined by orange dashed line. Scale bar 50µm. (I) focal adhesions color-coded according to size. (II) classification of focal adhesions according to size. **(c)** Percent of medium sized focal adhesions (1-5µm²). **(d)** Percent of small sized focal adhesions (0.25-1µm²). **(e)** Percent of tiny focal adhesions (<0.25µm²). **(f)** Gene expression of (from left to right): alpha 5 integrin (ITGA5), alpha V integrin (ITGAV), beta 5 integrin (ITGB5) 3 and 14 days post induction of cellular senescence (n=5-6).


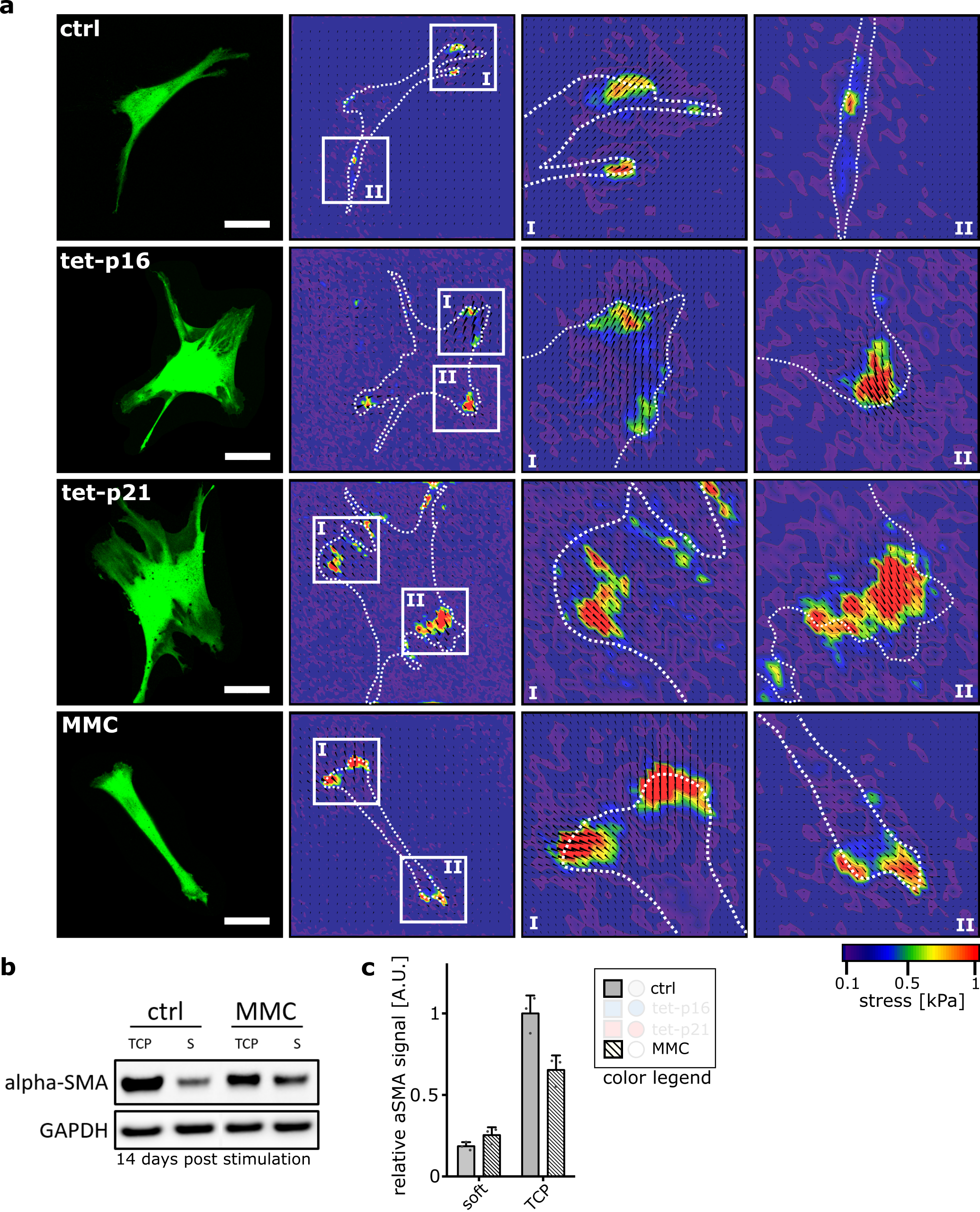


**Figure S3: representative heat maps for all groups. (a)** Left: cells stained with cell tracker green to visualize cell outline. Scale bar 50µm. Heat maps illustrate local stress scaled between 0.1 and 1kPa with overlaid displacement vectors (black arrows). White dashed lines illustrate cell outline. ROIs I and II illustrate zoom-in maps of cell adhesion points. **(b)** Representative Western Blot of cell cultured for 14 days either on plastic (TCP) or soft PDMS substrate (S). **(c)** Quantification of alpha-SMA signals after 14 days of culture either on 1kPa PDMS substrate (soft) or plastic (TCP). N=3


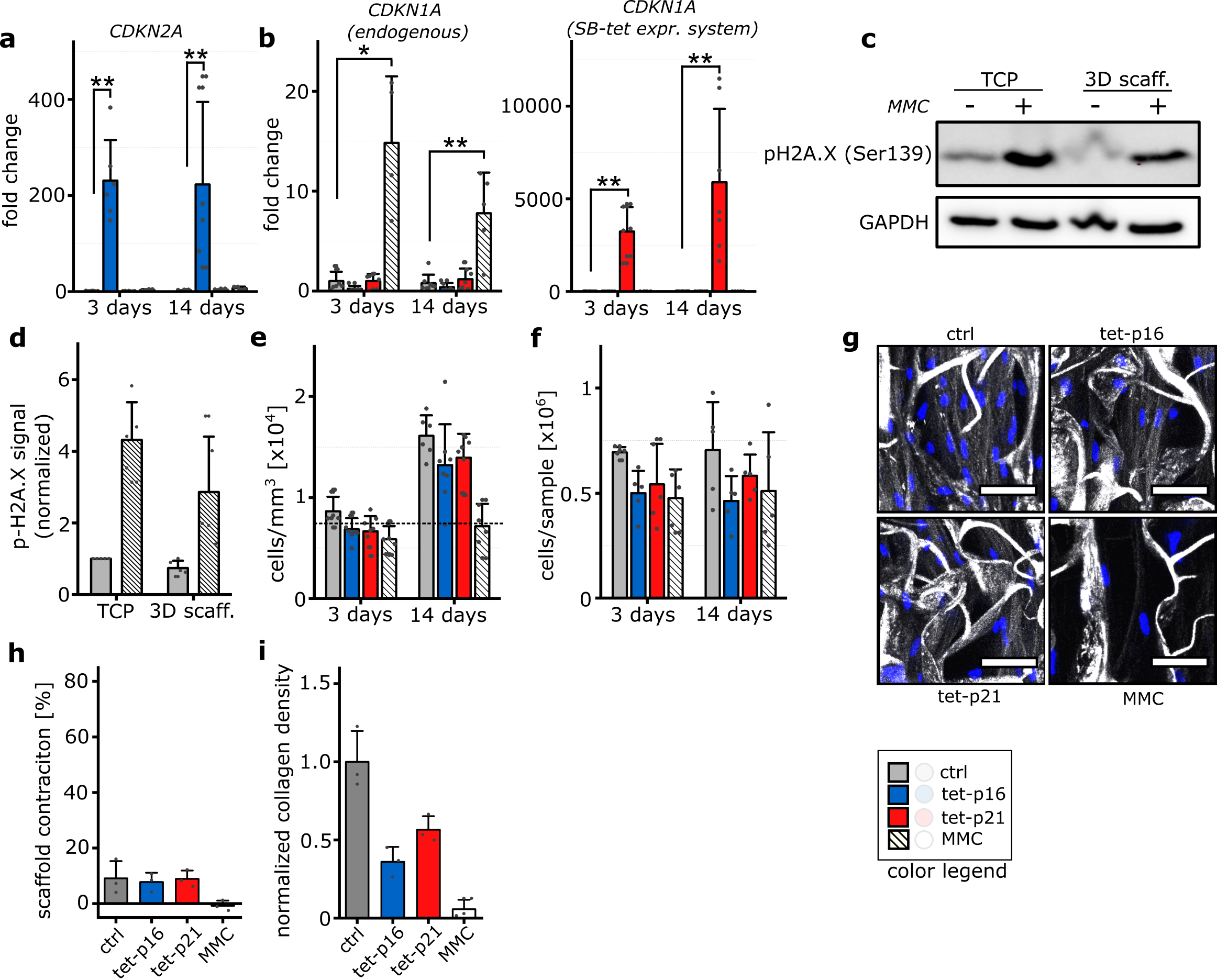


**Figure S4: (a)** Gene expression 3 and 14 days post induction of cellular senescence of p16 (CDKN2A), **(b)** of p21 (CDKN1A) either endogenous (left) or tet-inducible (right). **(c)** Representative western blot of phospho-H2A.X 24h after stimulation either in 2D (TCP) or inside 3D scaffolds. **(d)** Quantification of phospho-H2A.X levels (normalized to GAPDH). **(e)** Cell density inside scaffolds after 3 and 14 days of culture. Black dashed line illustrates initial seeding density of 7500cells/µl. **(f)** Total cell count per sample 3 and 14 days of culture (concentration x scaffold volume). **(g)** Confocal images illustrate cell-derived fibrillar collagen after 14 days of culture. Fibrillar collagen (white) was visualized by SHI, cell nuclei were stained and visualized in blue. Scale bar 50µm. **(h)** Scaffold contraction of stiff materials (3.0% solid content) after 14 days of culture. **(i)** Collagen density (normalized to control) after 14 days of culture for stiff scaffolds. N=3


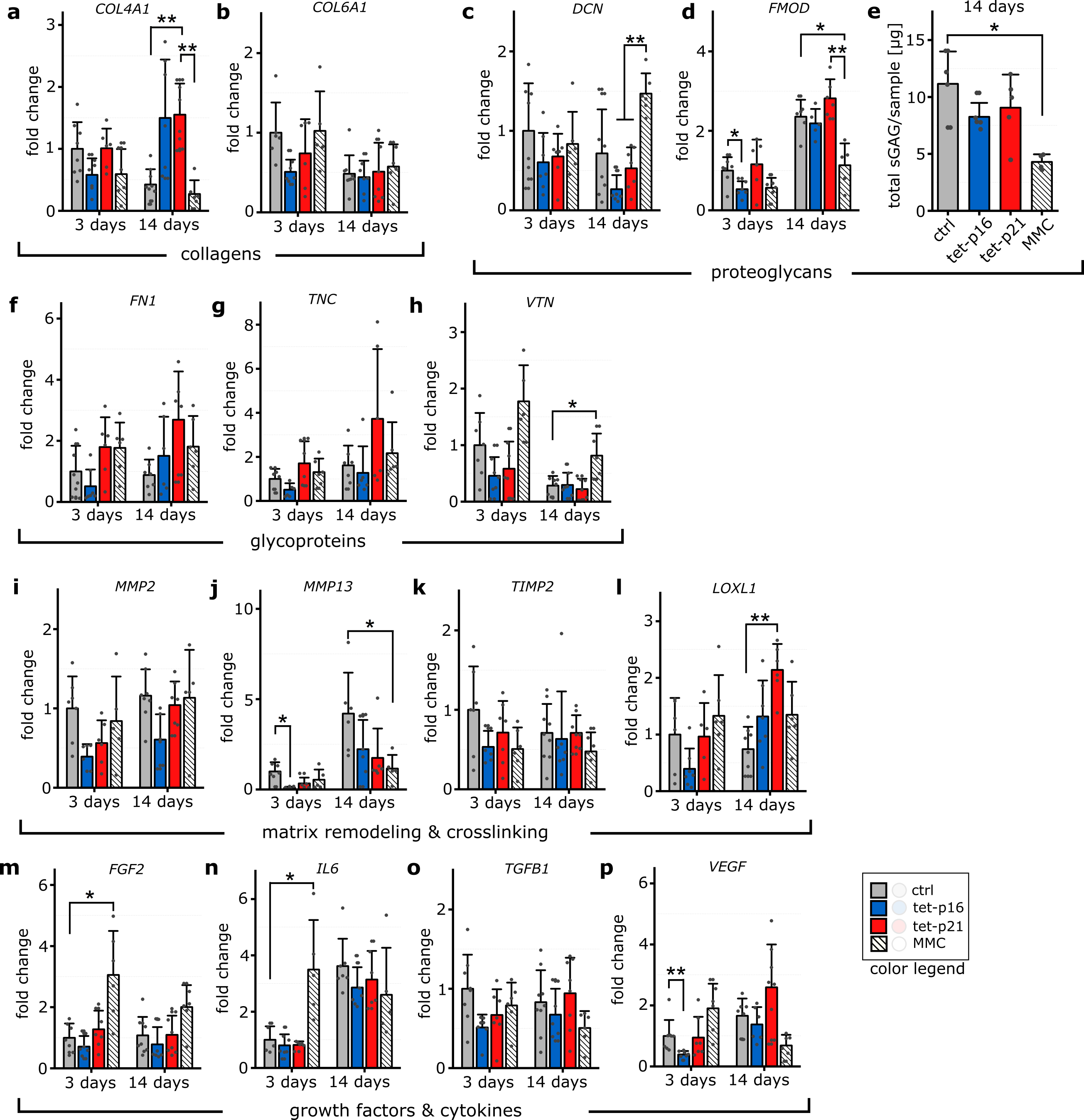


**Figure S5: (a-d, f-p)** Gene expression analysis of various ECM-related proteins including collagens (collagen type IV A1, type VI A1) proteoglycans (decorin, fibromodulin), glycoproteins (fibronectin, tenascin C, vitronectin), MMPs & TIMPS (matrix metalloproteinase 2, 13, tissue inhibitor of metalloproteinase 2, lysyl oxidase like 1) and SASP-related growth factors and cytokines (fibroblast growth factor 2, interleukin 6, transforming growth factor beta 1 and vascular endothelial growth factor A) 3 and 14 days post induction of cellular senescence. N=5-6 **(e)** sulfated glycosaminoglycan (sGAG) content per sample 14 days post induction of cellular senescence. N=4


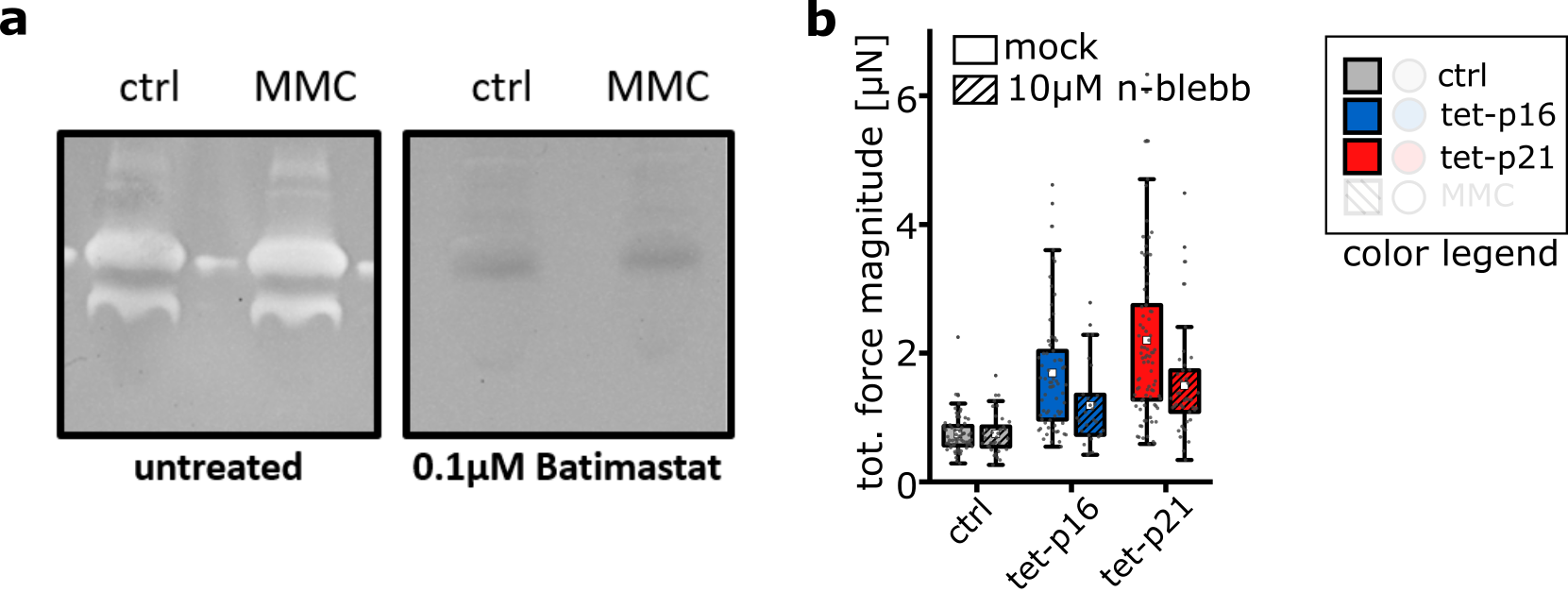


**Figure S6: (a)** Zymogram loaded with 5x conditioned media after 7days of culture for control and MMC-treated groups. 0.1µM Batimastat were used during gel incubation to demonstrate the efficacy of the inhibitor. White bands hereby indicate collagenase activity. **(b)** Total force magnitude (µN) detected for single cells either untreated (mock) or with 10µM nitro-blebbistatin for at least 2h (N>50).

**Supplementary Table 1:** Primers for gene expression analysis

| **Gene** | **Protein** | **Category** | **Primer Sequences 5'🡪 3'** | |
| --- | --- | --- | --- | --- |
|  |  |  | **forward (fwd) and reverse (rev)** | |
| *ACTA2* | Alpha-SMA | Cell mechanics | fwd | CTACGTGGGTGACGAAGCAC |
|  |  |  | rev | TGCTCTTAGGGGCAACACG |
| *B2MG* | Beta-2-microglobulin | Housekeeper | fwd | GGTTTCATCCATCCGACATT |
|  |  |  | rev | CGGCAGGCATACTCATCTTT |
| *CDKN1A* | Cyclin-dependent kinase inhibitor 1A, p21 | Cell cycle | fwd | ACAGCAGAGGAAGACCATGTG |
|  |  |  | rev | CTTCCTGTGGGCGGATTAGG |
| *CDKN1A* (tet-ind. construct) | Cyclin-dependent kinase inhibitor 1A, p21 | Cell cycle | fwd | TGGACTACAAAGACGATGACGA |
|  |  |  | rev | GTTCTGACGGACATCCCCAG |
| *CDKN2A* | Cyclin-dependent kinase inhibitor 2A, p16 | Cell cycle | fwd | CAACGCACCGAATAGTTACG |
|  |  |  | rev | ACCAGCGTGTCCAGGAAG |
| *COL1A2* | Collagen alpha-2(I) chain | ECM proteins | fwd | AGCCGGAGATAGAGGACCAC |
|  |  |  | rev | GGCCAAGTCCAACTCCTTTT |
| *COL3A1* | Collagen alpha-1(III) chain | ECM proteins | fwd | GGGAGAAATGGTGACCCTGG |
|  |  |  | rev | TGCGAGTCCTCCTACTGCTA |
| *ITGA1* | Integrin alpha 1 | Cell adhesion | fwd | ACGCTGCTGCGTATCATTCA |
|  |  |  | rev | CACCTCTCCCAACTGGACAC |
| *ITGA5* | Integrin alpha 5 | Cell adhesion | fwd | TGGCCTTCGGTTTACAGTCC |
|  |  |  | rev | GGTGCAGTTGAGTCCCGTAA |
| *ITGAV* | Integrin alpha V | Cell adhesion | fwd | TCAGCAAGGCAATGCTCCAT |
|  |  |  | rev | GAGGGCAAGATCCCGCTTAG |
| *ITGB1* | Integrin beta 1 | Cell adhesion | fwd | CTGCGAGTGTGGTGTCTGTA |
|  |  |  | rev | CACAGGATCAGGTTGGACCG |
| *ITGB3* | Integrin beta 3 | Cell adhesion | fwd | ACCAGTAACCTGCGGATTGG |
|  |  |  | rev | TCCGTGACACACTCTGCTTC |
| *ITGB5* | Integrin beta 5 | Cell adhesion | fwd | ATACCTGGAACAACGGTGGAG |
|  |  |  | rev | AGATCCTCAGGCTGATCCCA |
| *COL4A1* | Collagen alpha-1(IV) chain | ECM proteins | fwd | GCTTACCAGGGATGAAGGGTG |
|  |  |  | rev | TCACCTTTCAACAGCATCCCG |
| *COL5A1* | Collagen alpha-1(V) chain | ECM proteins | fwd | TCAATGGCATCATCGTGTTT |
|  |  |  | rev | GTGTCAGGTACTGCGGTGTC |
| *COL6A1* | Collagen alpha-1(VI) chain | ECM proteins | fwd | ACTGCGTATCAAGAAGGGG |
|  |  |  | rev | TCGTTCACAGCATCCTCCAG |
| *COL8A1* | Collagen alpha-1(VIII) chain | ECM proteins | fwd | ACCAGCAAGGACATTGGTCT |
|  |  |  | rev | GCGGCTTGATCCCATAGTAG |
| *DCN* | Decorin | ECM proteins | fwd | TTCCTGATGACCGCGACTTC |
|  |  |  | rev | CAGGGGGAAGATCCTTTGGC |
| *FGF2* | Fibroblast growth factor 2 | Cytokines and growth factors | fwd | AGCGGCTGTACTGCAAAAAC |
|  |  |  | rev | AGCCAGGTAACGGTTAGCAC |
| *FMOD* | Fibromodulin | ECM proteins | fwd | CAGCCTCCTTGAGCTAGACC |
|  |  |  | rev | GCAGCTTGGAGAAGTTCACG |
| *FN1* | Fibronectin | ECM proteins | fwd | CAGCCAGTAGCTTTGTGGTC |
|  |  |  | rev | GCATCAGGCGCTGTTGTTT |
| *IL-6* | Interleukin-6 | Cytokines and growth factors | fwd | AAAGAGGCACTGGCAGAAAA |
|  |  |  | rev | AGCTCTGGCTTGTTCCTCAC |
| *LOXL1* | Lysyl oxidase homolog 1 | ECM regulation | fwd | TGTACCGGCCCAACCAGAAC |
|  |  |  | rev | GATGCTTGCACATAGTTGGGG |
| *LOXL2* | Lysyl oxidase homolog 2 | ECM regulation | fwd | TGTGCAGCGACAAAAGGATTC |
|  |  |  | rev | CGCTTGCGGTAGGTTGAGAG |
| *MMP1* | Interstitial collagenase | ECM regulation | fwd | ACATGAGTCTTTGCCGGAGG |
|  |  |  | rev | ATCCCTTGCCTATCCAGGGT |
| *MMP2* | 72 kDa type IV collagenase | ECM regulation | fwd | GGGCATTCAGGAGCTCTATGG |
|  |  |  | rev | CAGTCCGCCAAATGAACCG |
| *MMP8* | Neutrophil collagenase | ECM regulation | fwd | AACCAGCAACTACTCACTCCC |
|  |  |  | rev | GTGCTTGGTCCAGTAGGTTG |
| *MMP13* | Collagenase 3 | ECM regulation | fwd | TTGAGCTGGACTCATTGTCG |
|  |  |  | rev | TCTCGGAGCCTCTCAGTCAT |
| *PDGFA* | Platelet derived growth factor A | Cytokines and growth factors | fwd | GCAACACGAGCAGTGTCAAG |
|  |  |  | rev | GCTCCTCTAACCTCACCTGG |
| *TGFB1* | Transforming growth factor beta 1 | Cytokines and growth factors | fwd | GGCCTTTCCTGCTTCTCAT |
|  |  |  | rev | GTCCTTGCGGAAGTCAATGT |
| *TIMP1* | Metalloproteinase inhibitor 1 | ECM regulation | fwd | GCTTCTGGCATCCTGTTGTT |
|  |  |  | rev | ACGCTGGTATAAGGTGGTCTG |
| *TIMP2* | Metalloproteinase inhibitor 2 | ECM regulation | fwd | CAAAGGGCCTGAGAAGGATA |
|  |  |  | rev | AGGCTCTTCTTCTGGGTGGT |
| *TNC* | Tenascin | ECM proteins | fwd | GTGAAAAACAATACCCGGGGC |
|  |  |  | rev | CCGTAGGTCAGCTCAATGCC |
| *VEGFA* | Vascular endothelial growth factor A | Cytokines and growth factors | fwd | CAGAAGGAGGAGGGCAGAAT |
|  |  |  | rev | CTGCATGGTGATGTTGGACT |
| *VTN* | Vitronectin | ECM proteins | fwd | GCTGGGAGGACATCTTCGAG |
|  |  |  | rev | ATGCCTGAGATGTAGATGCGG |
